# Guggulsterone Induces Apoptosis in Multiple Myeloma Cells by Targeting High Mobility Group Box 1 via Janus Activated Kinase/Signal Transducer and Activator of Transcription Pathway

**DOI:** 10.3390/cancers14225621

**Published:** 2022-11-16

**Authors:** Sabah Akhtar, Lubna Zarif, Shilpa Kuttikrishnan, Kirti S. Prabhu, Kalyani Patil, Sabah Nisar, Haissam Abou-Saleh, Maysaloun Merhi, Said Dermime, Ajaz A. Bhat, Shahab Uddin

**Affiliations:** 1 Translational Research Institute, Academic Health System, Hamad Medical Corporation, Doha 3050, Qatar; 2 Biological Science Program, Department of Biological and Environmental Sciences, College of Arts and Sciences, Qatar University, Doha 2713, Qatar; 3 Department of Pharmaceutical Sciences, College of Pharmacy, QU Health, Qatar University, Doha 2713, Qatar; 4 Department of Human Genetics, Sidra Medicine, Doha 26999, Qatar; 5 Biomedical Research Center, Qatar University, Doha 2713, Qatar; 6 National Center for Cancer Care and Research, Hamad Medical Corporation, Doha 3050, Qatar; 7 Dermatology Institute, Academic Health System, Hamad Medical Corporation, Doha 3050, Qatar; 8 Laboratory of Animal Research Center, Qatar University, Doha 2713, Qatar

**Keywords:** guggulsterone, multiple myeloma, apoptosis, JAK/STAT signaling, anti-apoptotic proteins, HMGB1

## Abstract

**Simple Summary:**

Multiple myeloma (MM) is a cancer of white blood cells known as plasma cells. It is hard to treat cancer, thus requires new treatments. Herein, a plant extracted compound, guggulsterone (GS), has been investigated for its anticancer activity in MM cells. The results from this study revealed that GS could be used for the effective treatment of MM due to its ability to cause cell death in MM cells. It exhibits anticancer activity itself and also increases the effectiveness of other drugs when combined. Therefore, it could be further investigated for its possible utilization in clinics to treat MM patients.

**Abstract:**

Multiple myeloma (MM) is a hematological disorder characterized by the abnormal expansion of plasma cells in the bone marrow. Despite great advances over the past three decades in discovering the efficacious therapies for MM, the disease remains incurable for most patients owing to emergence of drug-resistant cancerous cells. Guggulsterone (GS), a phytosteroid, extracted from the gum resin of guggul plant, has displayed various anticancer activities in vitro and in vivo; however, the molecular mechanisms of its anticancer activity have not been evaluated in MM cells. Therefore, in this study, we investigated the anticancer activity of GS in various MM cell lines (U266, MM.1S, and RPMI 8226) and the mechanisms involved. GS treatment of MM cells caused inhibition of cell proliferation and induction of apoptotic cell death as indicated by increased Bax protein expression, activation of caspases, and cleavage of poly (ADP-ribose) polymerase. This was associated with the downregulation of various proliferative and antiapoptotic gene products, including cyclin D, Bcl-2, Bcl-xL, and X-linked inhibitor of apoptosis protein. GS also suppressed the constitutive and interleukin 6-induced activation of STAT3. Interestingly, the inhibition of Janus activated kinase or STAT3 activity by the specific inhibitors or by siRNA knockdown of STAT3 resulted in the downregulation of HMGB1, suggesting an association between GS, STAT3, and HMGB1. Finally, GS potentiated the anticancer effects of bortezomib (BTZ) in MM cells. Herein, we demonstrated that GS could be a potential therapeutic agent for the treatment of MM, possibly alone or in combination with BTZ.

## 1. Introduction

Multiple Myeloma (MM) is a rare form of hematologic malignancy that is characterized by excessive proliferation and accumulation of malignant clonal plasma cells in the bone marrow microenvironment, resulting in anemia, hypercalcemia, extensive skeletal destruction, abnormal bleeding, and renal failure [1,2]. Over the past three decades, advances in treatment regimens have dramatically improved life quality and expectancy in MM patients. However, the emergence of drug-resistant clones has made the disease incurable in most patients, leading to worse survival outcomes. Therefore, there is a dire need to develop novel and innovative strategies that lead to effective therapies against this dreadful disease [1,3].

Various studies have revealed the involvement of Janus-activated kinase (JAK)/signal transducer and activator of transcription (STAT) signaling pathway in the pathogenesis of MM. The JAK/STAT pathway is a pleiotropic signaling cascade and a key player in various physiological processes, including immune function, hematopoiesis, cell growth, and apoptosis [4]. Accumulating evidence has shown the dysregulation of the JAK/STAT signaling pathway in various cancers, thus putting the JAK/STAT pathway members as potential targets for anticancer therapeutic development [5]. Numerous solid cancers, such as breast, colon, and cervical cancers, exhibit increased activation of STAT3 [6]. The dysregulation of JAK/STAT signaling pathway has been described in various hematological malignancies, particularly in MM. The aberrant activation of the JAK/STAT signaling pathway in MM is initiated by interleukin 6 (IL-6)-induced phosphorylation of STAT in the bone marrow [7]. Indeed, it has been demonstrated that MM cells depend on cytokine stimulation, especially IL-6, for their continuous growth [8]. Studies have revealed that MM patients often exhibit high levels of activated STAT3 [7]. The JAK/STAT signaling pathway has been shown to modulate the expression of the high mobility group box 1 (HMGB1) in various malignancies [9]. The activation of the JAK/STAT signaling pathway regulates the nuclear-to-cytoplasmic translocation of HMGB1 and its subsequent release in the extracellular space [9].

Compelling evidence has indicated the association of HMGB1 with various hallmarks of cancer, such as angiogenesis, apoptosis, tissue invasion, metastasis, inflammation, and insensitivity to growth inhibitors [10,11]. In MM, HMGB1 has been shown to induce chemoresistance through the *nuclear factor-κB* pathway, evidenced by the increased sensitivity to chemotherapy and induction of DNA damage and cellular apoptosis upon downregulation of HMGB1 [12,13]. In patients with MM, HMGB1 has been reported to be negatively associated with patient survival at 3 years [12]. Collectively, these studies indicate the emergent oncogenic role of HMGB1 and its importance as a critical molecular target in MM drug resistance. Nevertheless, further investigations are warranted to understand the role and regulatory mechanism of HMGB1 in MM.

**Figure 1:**
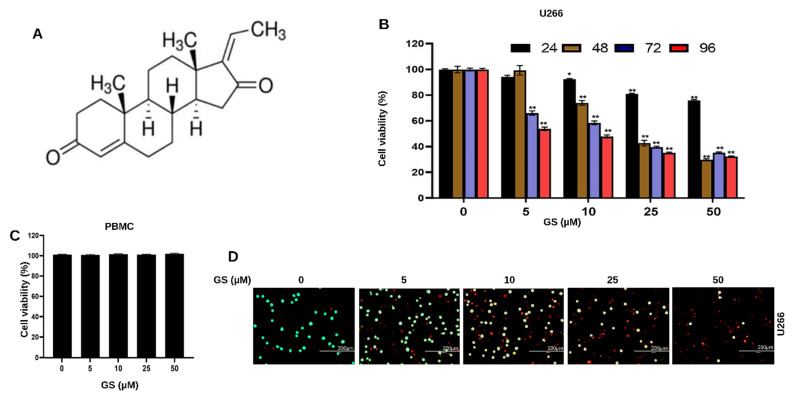
Anti-proliferative effects of GS on MM cells. (**A**) Chemical structure of Z-isomer of guggulsterone (4,17(20)-pregnadiene-3,16-dione). GS inhibits the viability of (**B**) U266 in a concentration-dependent manner but does not inhibit the viability of (**C**) PBMCs (normal control cells) after treatment with 5, 10, 25, and 50 µM of GS for 48 h. The graphs display the mean ± SD of three independent experiments with replicates. * *p* < 0.05, ** *p* < 0.001. (**D**) Cell viability assessment performed using live/dead cytotoxicity kit at 5, 10, 25, and 50 µM GS treatment on U266 cells where green indicates live cells and red indicates non-viable cells (Scale bar, 200 μm).

Guggulsterone (GS, 4,17(20)-pregnadiene-3,16-dione, Figure 1A), is a phytosteroid found in the gum resin of the guggul plant, *Commiphora mukul* (Hook. Ex Stocks) Engl., also known as *Commiphora wightii* (Arn.) Bhandari [14]. Owing to its potent anti-inflammatory, antioxidant, hypolipidemic, and hypoglycemic activities, GS has been effectively used in Ayurvedic medicine to treat a wide variety of conditions, such as obesity, diabetes, cardiovascular disease, osteoarthritis, inflammation, hyperlipidemia, and solid tumors [15,16,17,18]. GS has been shown to be orally active in animal studies; in rats, it has an absolute bioavailability of 42.9% following oral administration and a half-life of approximately 10 h, indicating a favorable pharmacokinetic profile. In subchronic and chronic toxicity trials, rats, dogs, or rhesus monkeys were given a standardized guggul extract (125–500 mg/kg) for 90–180 days without experiencing any side effects. Dogs who received 1 g of guggul extract daily for 3 months showed no signs of death [19]. However, among the most often reported side effects in humans was gastrointestinal distress [20,21,22]. However, the toxicity and bioavailability studies of GS have not been performed yet in cancer clinical studies. Therefore, this is an area of future investigation. Besides GS, a structurally similar compound to GS, β-sitosterol (phytosterol), has displayed anticancer potential in MM [23]. In addition to beta-sitosterol, some other phytochemicals have also been explored for their anticancer potential for MM, such as genipin, compound K, and icariside II [24,25,26]. One of the common mechanisms that GS employs to induce apoptosis in various solid tumors is through its pro-apoptotic activity, generation of reactive oxygen species, downregulation of antiapoptotic proteins, modulation of cell cycle proteins, and activation of caspases [27,28]. Ahn and colleagues have elucidated the anticancer potential of GS against MM cells, demonstrating that GS suppresses STAT3 activation by inducing the expression of protein tyrosine phosphatase (PTP), SH2 domain-containing phosphatase 1 SHP-1 [29]. However, there is a gap in the literature regarding the association of the JAK/STAT pathway with HMGB1 in MM drug resistance. Moreover, there is a lack of knowledge about the combinational treatment effect of GS with other drugs. Therefore, the current study aims to unravel the molecular mechanisms that underscore the anti-proliferative effects of GS in MM cells and, most importantly, to explore the effect of GS alone or in combination with bortezomib (BTZ), on regulating the JAK/STAT signaling pathway and association of this pathway with the expression of HMGB1. This can help to delineate the mechanisms underlying the survival and chemoresistance of MM cells, which will eventually help to identify molecular targets to treat this malignancy.

## 2. Materials and Methods

### 2.1. Reagents and Antibodies

The human MM cell lines MM.1S, RPMI 8266, and U266-B1 were procured from the American-type culture collection (ATCC, Manassas, VA, USA). Fetal bovine serum (FBS), penicillin-streptomycin (PenStrep), and RPMI-1640 media were obtained from Thermo Fisher Scientific (Waltham, MA, USA). Z-GS and JAK inhibitor AG-490 were purchased from Tocris Bioscience (Bristol, UK) and were prepared in dimethyl sulfoxide (DMSO). Z-VAD-FMK was purchased from Calbiochem (San Diego, CA, USA). Cell counting kit-8 (CCK-8) and N-acetylcysteine were purchased from Sigma Aldrich (St. Louis, MO, USA). Live/dead cell viability/cytotoxicity kit was purchased from Molecular Probes (Eugene, OR, USA). Annexin-V FITC apoptosis detection kit, fixation/permeabilization solution kit, JC-1, BV421 mouse anti-gamma H2AX (pS139), PE rabbit anti-active caspase-3 and Alexa Fluor 700 mouse anti-cleaved PARP (Asp214) were obtained from BD Biosciences (Franklin Lakes, NJ, USA). The HMGB1 ELISA kit was purchased from Biomatik (Cambridge, ON, Canada). Caspase 3/7 green detection reagent was purchased from Invitrogen (Waltham, MA, USA). STAT3 siRNA was purchased from Life Technologies (Carlsbad, CA, USA), and control siRNA was purchased from Qiagen (Hilden, Germany). Antibodies such as p-H2AX, cytochrome c, caspase-3, cleaved caspase-3, caspase-9, PARP, Bcl-2, Bax, Bcl-xL, cyclin D1, LC3, Atg7, Atg5, p62, STAT3, p-STAT3, SHP-1, and HMGB1 were purchased from Cell Signaling Technology (Danvers, MA, USA). Other antibodies, such as BID, XIAP, p21, and p27, were purchased from Abcam (Cambridge, UK). Loading control antibodies, such as GAPDH, β-actin, and HSP60, were obtained from Santa Cruz Biotechnology (Santa Cruz, CA, USA).

### 2.2. Cell Culture

MM cells were cultured in RPMI 1640 medium supplemented with 20% FBS, 1% L-glutamine, 1% penicillin-streptomycin and incubated with 5% CO_2_ at 37 °C. Peripheral blood mononuclear cells (PBMCs) were obtained from healthy volunteers and were maintained under the same conditions as MM cells.

### 2.3. Cell Viability Assay

Human MM.1S, U266-B1, and RPMI 8266 (10,000 cells/well) were seeded in 96-well plates and treated with increasing concentrations of GS (5, 10, 25, and 50 µM). The cells were then incubated for 24, 48, 72, and 96 h. After the completion of the time point, 10 µL of CCK-8 reagent was added to the cells and the plate was incubated for 1 h at 37 °C. The absorbance was measured at 450 nm, and the number of viable cells was estimated as described previously [30].

### 2.4. Live/Dead Assay

U266 cells were seeded at a density of 5 × 10^3^ cells/well in a 6-well plate and treated with GS for 48 h. Live/dead stain was prepared by adding 5 μL of ethidium homodimer-1 (EthD-1) and 5 μL of calcein AM to 10 mL PBS (i.e., final concentrations of 1 μM EthD-1 and 2 μM calcein). The cells were stained with the prepared dye for 30 min and then images were captured using the EVOS FLoid cell imaging system (Thermo Fisher Scientific, Waltham, MA, USA).

### 2.5. Caspase-3/Caspase-7 Green Apoptosis Assay

U266 cells (at a seeding density of 5 × 10^3^) were treated with GS for 48 h in a 12-well plate. After the treatment time point was completed, caspase-3/caspase-7 green detection reagent was added to the cells, and the cells were incubated for 30 min at 37 °C. The cells were then visualized, and images were captured using the EVOS FLoid cell imaging system (Thermo Fisher Scientific, Waltham, MA, USA).

### 2.6. Cell Lysis and Immunoblotting

U266 cells were collected after 48 h of GS treatment and lysed with 2× laemmli sample buffer. The proteins were quantified using ND-100 Nanodrop (Thermo Fisher Scientific, Waltham, MA, USA), and β-mercaptoethanol was added. SDS-PAGE technique was used to separate protein cell lysates (30–60 µg), which were transferred onto the polyvinylidene difluoride (PVDF) membrane. The membranes were immunoblotted with antibodies and developed on the ChemiDoc imaging system (Bio-Rad, Hercules, CA, USA).

### 2.7. AnnexinV/Propidium Iodide (PI) Dual Staining

U266 cells (at a seeding density of 1 × 10^6^) were incubated with various concentrations of GS for 48 h. The cells were collected in 1× annexin binding buffer after washing with PBS and stained with Annexin V-FITC and PI for 20 min. The cells were analyzed and quantified as live (Annexin FITC^−ve^, PI^−ve^), early apoptotic (Annexin FITC^+ve^, PI^−ve^), late apoptotic (Annexin FITC^+ve^, PI^+ve^), and necrotic (Annexin FITC^−ve^, PI^+ve^) using BD LSRFortessa cell analyzer (BD Biosciences, Franklin Lakes, NJ, USA). Total apoptosis percentages were measured by adding early and late apoptosis percentages [31].

### 2.8. Measurement of DNA Double-Strand Breaks

To measure DNA double-strand breaks, U266 cells (1 × 10^5^) were fixed and permeabilized using a permeabilization and fixation kit and stained with 5 µL H2AX (pS139)-Alexa Fluor 647 antibody. After staining, the cells were then analyzed using the BD LSRFortessa cell analyzer (BD Biosciences, Franklin Lakes, NJ, USA).

### 2.9. Measurement of Mitochondrial Membrane Potential

To determine the effect of GS treatment on the mitochondrial activity of MM cells, the JC1 stain kit was used as previously described [32]. Cells were stained with JC1 stain in the dark, and MMP was determined using the BD LSRFortessa cell analyzer (BD Biosciences, Franklin Lakes, NJ, USA). The mean values that represented the loss of MMP representation were plotted in a bar graph.

### 2.10. Flow Cytometric Analysis of Activated Caspase-3 and Cleaved PARP

U266 cells (5 × 10^5^) were fixed, permeabilized, and stained with AF700 tagged cleaved PARP, and BV605 tagged active caspase-3 for 30 min. The analysis was carried out via flow cytometry, as previously described [32].

### 2.11. HMGB1 ELISA

U266 cells were collected after treatment with GS and centrifuged. The cell supernatant was collected for the experiment. The HMGB1 ELISA assay was performed using the human HMBG-1 ELISA kit (LSBio, Seattle WA, USA).

### 2.12. Gene Silencing Using siRNA

4D-Nucleofactor^TM^ System (Lonza) was used for the transfection of U266 cells with control siRNA (Qiagen, Hilden, Germany) and STAT3 siRNA (Life Technologies, Carlsbad, CA, USA) according to the manufacturer’s protocol. After transfection, the cells were incubated for 48 h at 37 °C and then lysed and immunoblotted with different antibodies.

### 2.13. Multiplex Cytokine Analysis

Cytokine measurement was performed using a multiplexing array BD^TM^ Cytometric Bead Array kit (BD Biosciences). The supernatant of U266 was collected, and IL-6 levels were measured. In tubes containing cytokine standards, detection antibodies were added and after 2 h incubation, Streptavidin-PE was added to the mix and subsequently washed with Tween 20 and PBS (PBS/T). After an additional 1 h incubation, the samples were washed with PBS/T, and the beads were resuspended in PBS with 10% FBS. The samples were analyzed by a BD LSRFortessa cell analyzer (BD Biosciences, Franklin Lakes, NJ, USA).

### 2.14. Statistical Analysis

The statistical analysis was performed using GraphPad Prism software version 7.0 (San Diego, CA, USA). One-way analysis of variance (ANOVA) was utilized to evaluate the statistical differences between control and treatment groups, followed by Sidak’s post hoc test. Results are presented as mean ± standard deviation (SD). The differences between groups were considered statistically significant at *p* ≤ 0.05.

## 3. Results

### 3.1. GS Treatment Reduces the Viability of MM Cells

To explore the effect of GS on the viability of MM cells, U266, MM.1S, and RPMI 8226 cells were treated with different concentrations of GS and incubated for 24, 48, 72, and 96 h. CCK-8 solution was used to assess cell viability. Figure 1B shows the effect of GS treatment on U266 cells at 24, 48, 72, and 96 h. A significant reduction was observed in the cell viability of U266 cells in a concentration-dependent manner (Figure 1B). The same pattern was observed in MM.1S (Supplementary Figure S1A) and RPMI 8226 cells (Supplementary Figure S1B). At 24 h, the half-maximal inhibitory concentration (IC_50_) was not achieved for all three cell lines. However, at 48 h, IC_50_ for U266 and RPMI 8226 cells was observed at 11.9 µM and 35.6 µM (Supplementary Figure S2A,D), whereas it was not achieved for MM.1S. IC_50_ values for all three cell lines were observed at 72 h, which were 12.4 µM, 23.9 µM, and 32.4 µM for U266, RPMI 8226, and MM.1S, respectively (Supplementary Figure S2B,E,G), and at 96 h, the IC_50_ values were 6.9 µM, 20.2 µM and 26.1 µM for U266, RPMI 8226 and MM.1S, respectively (Supplementary Figure S2C,F,H). The optimal GS treatment period for this study was 48 h, and U266 and RPMI 8226 cells were chosen for the remainder of the experiments in the study. This is because these cells can be easily cultured, and GS treatment caused an appreciable cytotoxic effect on U266 and RPMI 8226 cells at 48 h. To determine the effects of GS on normal cells, PBMCs were treated with the same concentrations of GS mentioned above for 48 h, and the cell viability was assessed by adding CCK-8 solution to the cells. Results showed that GS has no cytotoxic effect on PBMCs as no difference was observed in the cell viability compared to untreated cells; hence, IC_50_ was not achieved (Figure 1C). In the next experiment, live/dead assay was performed. After 48 h of GS treatment, U266 cells were stained with 1 μM EthD-1 and 2 μM calcein AM and incubated for 30 min. The live cells stain green because Calcein AM easily enters the cells via diffusion and is converted to calcein by intercellular esterase. Meanwhile, dead or damaged cells stain red because the cells take up EthD-1. Images show a GS-mediated decrease in U266 viability with maximum death observed at 50 µM GS concentration (Figure 1D, Supplementary Figure S3A). The GS-mediated cell death is also confirmed in RPMI 8226 cells, where the number of non-viable cells increases upon increasing GS concentration (Supplementary Figure S3B).

### 3.2. GS Induces Apoptosis and Mediates Cellular DNA Damage in MM Cells

**Figure 2:**
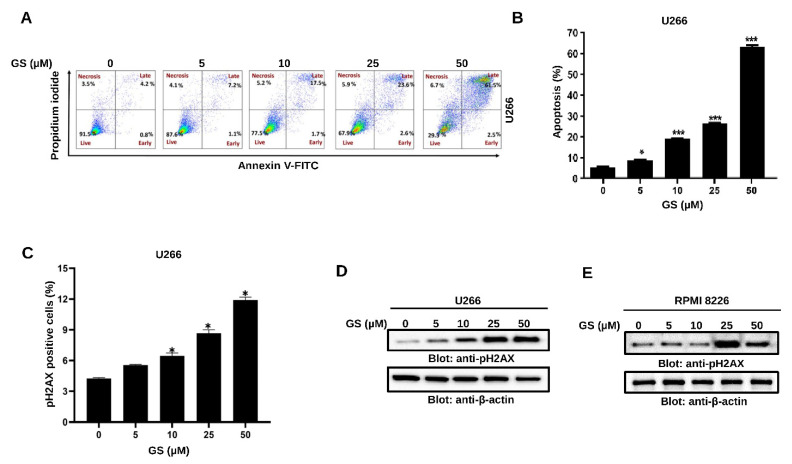
Apoptotic effects of GS on MM cells. (**A**) U266 cells were incubated with 5, 10, 25, and 50 µM of GS for 48 h. Representative graphs were obtained by flow cytometric analysis after double-staining with Annexin-V-FITC/PI. (**B**) The graph displays the values of mean ± SD of the percentage of the apoptotic cells of three independent experiments. * *p* < 0.05, *** *p* < 0.01. (**C**) U266 cells were incubated with 5, 10, 25, and 50 µM of GS for 48 h and analyzed for DNA double-stranded breaks using flow cytometry. The graph displays the mean ± SD of three independent experiments * *p* < 0.05. (**D**) U266 and (**E**) RPMI 8226 cells were treated with increasing concentrations of GS for 48 h, lysed, and immunoblotted against p-H2AX and β-actin. Full Western blot images can be found at Supplementary Materials File S1.

The GS-mediated reduction in cell viability prompted us to evaluate if the MM cells undergo apoptosis upon exposure to GS. Therefore, Annexin V-FITC/PI dual staining experiment was performed using flow cytometry. Cell membranes remain intact during early apoptotic stages, so cells stain positive for Annexin V-FITC but negative for PI. However, late apoptotic cells are double-positive, and cells in the necrotic stage stain negative for Annexin V-FITC and positive for PI due to the loss of plasma membrane integrity. The lower quadrants represent the live cell population (lower-left quadrant) and early apoptotic cell population (lower-right quadrant). In comparison, the upper quadrants represent the late apoptotic cell population (upper-right quadrant) and the necrotic cell population (upper-left quadrant) (Figure 2A). A concentration-dependent increase in apoptosis was observed upon treatment of U266 cells with increasing concentrations of GS (5, 10, 25, and 50 µM) (Figure 2B). GS-mediated apoptosis was further analyzed by DNA fragmentation, a hallmark of apoptosis. Therefore, we assayed the expression of p-H2AX at serine 139, which indicates a double-stranded break [33]. The bar graph in Figure 2C highlight the significant percentage of DNA breaks at a GS concentration of 10 µM and above. The GS-mediated phosphorylation of H2AX was further confirmed by Western blotting of the histone variant, p-H2AX, in U266 (Figure 2D) and RPMI 8226 (Figure 2E) cells. These results indicate that GS efficiently induces apoptotic cell death and DNA fragmentation in U266 and RPMI 8226 cells.

### 3.3. GS Activates the Intrinsic and Extrinsic Apoptotic Pathways in MM Cells

**Figure 3:**
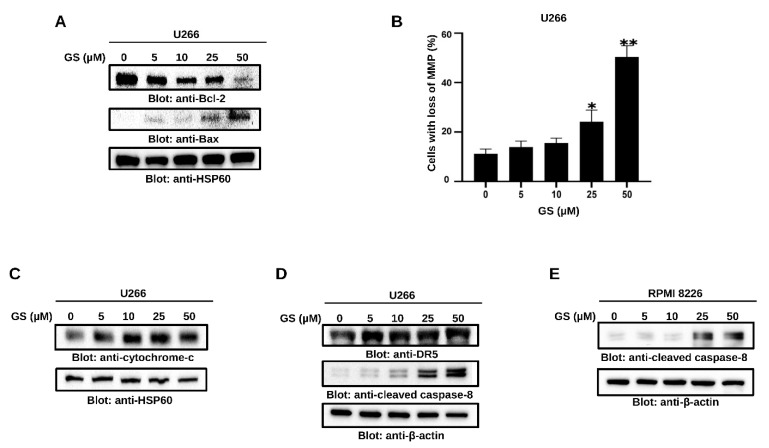
GS-mediated intrinsic and extrinsic apoptotic pathways in MM cells. U266 cells were treated with increasing concentrations of GS for 48 h, and cell lysates were immunoblotted with antibodies for (**A**) Bcl-2, Bax, and HSP60. (**B**) U266 cells were incubated with 5, 10, 25, and 50 µM of GS for 48 h, and cells were stained with JC1 stain and analyzed using flow cytometry. The graph displays the mean ± SD of three different experiments * *p* < 0.05 ** *p* < 0.01. U266 and RPMI 8226 cells were treated with increasing concentrations of GS for 48 h, and protein expression levels of (**C**) cytochrome c, HSP60. (**D**) DR-5, cleaved caspase-8, and β-actin in U266 cells and (**E**) cleaved caspase-8, and β-actin in RPMI 8226 cells were determined. Full Western blot images can be found at Supplementary Materials File S1.

The Bcl-2 family of proteins controls the intrinsic apoptotic pathway, primarily by affecting the mitochondrial membrane integrity. Under stress conditions, pro-apoptotic proteins of the Bcl-2 family promote the permeabilization of the mitochondrial outer membrane and the subsequent release of cytochrome c into the cytoplasm [34]. Our results showed that GS treatment of U266 cells decreased Bcl-2 and increased Bax protein expression levels (Figure 3A). Additionally, GS treatment of U266 cells caused a concentration-dependent loss of MMP (Figure 3B), causing the release of cytochrome c in U266 cells (Figure 3C). To further evaluate the effect of GS on the extrinsic apoptotic pathway, the expression of DR-5 was determined. Figure 3D shows an increased protein expression of DR-5 in U266 upon treatment with GS. Additionally, the protein expression of cleaved caspase-8 was increased in U266 (Figure 3D) and RPMI 8226 (Figure 3E) cells in a concentration-dependent manner, suggesting that GS-mediated apoptosis also involves the activation of the extrinsic apoptotic signaling pathway.

**Figure 4:**
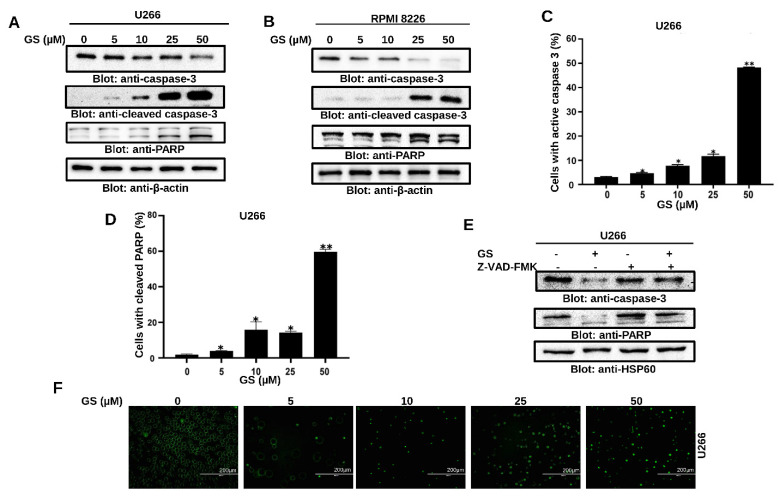
GS-mediated PARP cleavage and caspase activation in MM cells. U266 (**A**) and RPMI 8226 (**B**) cells were treated with 5, 10, 25, and 50 µM of GS for 48 h. 50 µg of proteins were resolved on SDS-PAGE and probed against caspase-3, cleaved caspase-3, PARP, and β-actin. Flow cytometric analysis of GS-mediated activation of caspase-3 (**C**) and cleavage of PARP (**D**) in U266 cells. The graph displays the mean ± SD of three different experiments (* *p* < 0.05, ** *p* < 0.01). U266 cells were treated with 30 µM z-VAD-FMK for 2 h, followed by 50 µM of GS for 48 h. Cells were lysed, and protein expressions of (**E**) caspase-3, PARP, and HSP60 were determined. (**F**) Representative fluorescence images reporting active caspase-3/caspase-7 (green nuclei) in apoptotic U266 cells (Scale bar, 200 μm). Full Western blot images can be found at Supplementary Materials File S1.

Next, to explore the effect of GS on the activation of caspase-cascades, U266 and RPMI 8226 cells were treated with increasing concentrations of GS and immunoblotted against caspase-3, cleaved-caspase-3, and PARP antibodies. Figure 4A,B show that treatment of U266 and RPMI 8226, respectively, with GS, resulted in the activation of caspase-3 and cleavage of PARP in a concentration-dependent manner. These results were further confirmed by flow cytometry (Figure 4C,D). z-VAD-FMK, a universal caspase inhibitor, prevented the GS-mediated caspase-3 activation and cleavage of PARP in U266 cells (Figure 4E). In addition, GS-mediated activation of caspase-3/caspase-7 was examined using the caspase-3/caspase-7 green detection reagent and analyzed using an epifluorescence microscope as described in the methods. Results showed that U266 cells treated with increasing concentrations of GS exhibited activation of caspase- 3/caspase-7 signals (Figure 4F). These results confirm that GS-mediated apoptosis involves the activation of caspase cascades.

### 3.4. GS Treatment Downregulates the Expression of HMGB1 via the JAK/STAT Pathway

**Figure 5:**
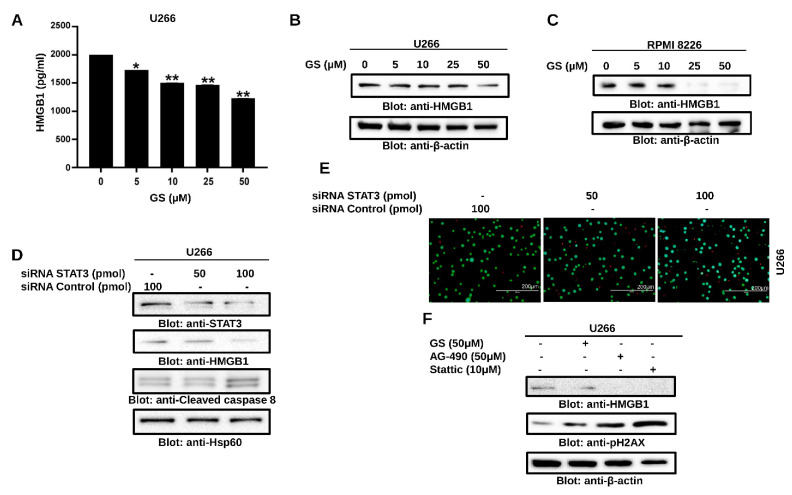
GS downregulates HMGB1 expression via the JAK/STAT signaling pathway. U266 cells were incubated with GS for 48 h and cell supernatant was collected and measured for (**A**) levels of HMGB1 using ELISA. The graph displays the mean ± SD of three independent experiments. * *p* < 0.05, ** *p* < 0.01. 50 µg of proteins from (**B**) U266 and (**C**) RPMI 8226 cells were resolved on SDS-PAGE, and probed against HMGB1 and β-actin. (**D**) U266 cells were transfected with either control (100 pM) or STAT3 specific siRNA (50 or 100 pM) and protein expression of STAT3, HMGB1, cleaved-caspase-8 and HSP60 was determined. (**E**) Cell viability was also determined using a live/dead cytotoxicity kit (Scale bar, 200 μm). (**F**) U266 cells were treated with 50 µM GS, 50 µM of AG-490 and 10 µM Stattic for 48 h. A total of 50 µg of proteins were separated on SDS-PAGE and protein expression of HMGB1, p-H2AX and β-actin were determined. Full Western blot images can be found at Supplementary Materials File S1.

To assess the role of HMGB1 in GS-treated U266 cells, HMGB1 ELISA was performed. Our results indicated a significant reduction in the secretion of HMGB1 in a concentration-dependent manner after 48 h of GS treatment (Figure 5A). In addition, Western blot analysis confirmed the downregulation of HMGB1 in MM cells after treatment with GS (Figure 5B,C). Gene silencing of STAT3 downregulated the expression of HMGB1 (Figure 5D), indicative of the JAK/STAT-mediated regulation of HMGB1. Furthermore, STAT3-mediated downregulation of HMGB1 caused activation of caspase-8 and induction of cell death in U266 cells (Figure 5D,E), indicating the role of the JAK/STAT/HMGB1 axis in apoptosis. Additionally, to understand the role of the JAK/STAT pathway in regulating the expression of HMGB1, U266 cells were incubated with GS and AG-490, a JAK2 inhibitor, and stattic, a small molecular weight inhibitor of STAT3, for 48 h. The results demonstrated that the expression of HMGB1 decreased upon treatment of U266 with GS, AG-490, and stattic (Figure 5F). Additionally, treating U266 cells with these compounds induced the expression of p-H2AX, an indicator of DNA damage/apoptosis.

### 3.5. GS Suppresses Constitutively Active and IL-6-Induced STAT3 Activation in MM Cells

**Figure 6:**
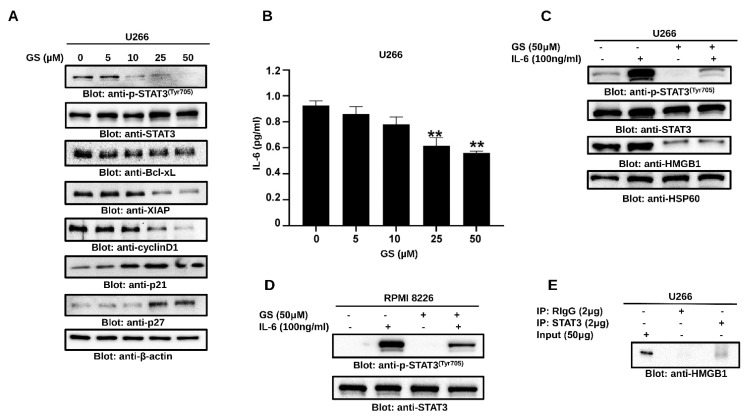
GS downregulates the constitutive and inducible STAT3 signaling. U266 cells were incubated with increasing concentrations of GS for 48 h, and (**A**) protein expressions of p-STAT3, STAT3, Bcl-xL, XIAP, Cyclin D1, p21^Cip1^ p27^Kip1^, and β-actin were determined. (**B**) GS-mediated inhibition of IL-6 secretion. GS treated U266 cells supernatant was collected, and quantitative analysis for IL-6 was performed using a multiplexing kit. The graph displays mean ± SD (n = 3 and ** *p* < 0.001). IL-6-induced STAT3 activation in MM cells; 24 h serum-starved (**C**) U266 and (**D**) RPMI 8226 cells were pre-treated with 50 μM GS for 48 h and then with IL-6 (100 ng/mL) for 30 min. Total proteins were extracted and probed against p-STAT3, STAT3, HMGB1, and HSP60. (**E**) Association of STAT3 and HMGB1. U266 cells were lysed and immunoprecipitated either with normal rabbit IgG (RIgG) or antibody against STAT3 using sepharose 4G. Immunoprecipitated proteins were analyzed by SDS-PAGE and immunoblotted with antibody against HMGB1. Full Western blot images can be found at Supplementary Materials File S1.

To further explore the effect of GS on the STAT3 signaling pathway, U266 cells were treated with GS and immunoblotted with antibodies against p-STAT3, STAT3, Bcl-xL, XIAP, cyclin D1, p21, and p27. GS treatment was found to inhibit the phosphorylation of STAT3 at Tyr705, in a concentration-dependent manner (Figure 6A) without affecting the total STAT3 protein levels (Figure 6A). Moreover, GS treatment downregulated the expression of Bcl-xL, XIAP and cyclin D1, and upregulated the expression of cell cycle inhibitors, p21 and p27, in a concentration-dependent manner (Figure 6A). Furthermore, our data showed that GS treatment upregulated the expression of SHP-1, (Supplementary Figure S3C) a PTP that negatively regulates the activation of STAT3 [35]. This effect was concurrent with the downregulation of phosphorylated STAT3 in MM cells.

IL-6 plays a vital role in sustaining the growth and proliferation of MM [36] via the activation of STAT3 [37]. Therefore, we measured the secretion of IL-6 in the culture media of U266 cells treated with GS, and the secretion of IL-6 was significantly reduced. Untreated cells exhibited a basal release of 0.95 pg/mL ± 0.03 of IL-6, while the concentration of 25 µM and 50 µM of GS decreased the IL-6 concentration to 0.61 ± 0.06 and 0.56 ± 0.01 pg/mL, respectively (Figure 6B). IL-6-treated U266 and RPMI 8226 cells showed an increased level of STAT3 phosphorylation, while those pre-treated with GS exhibited decreased STAT3 phosphorylation, suggesting that GS inhibits IL-6-induced STAT3 activation (Figure 6C,D). Interestingly, GS treatment of U266 cells also led to the downregulation of HMGB1, suggesting the involvement of HMGB1 in IL-6-mediated STAT3 signaling in MM cells (Figure 6C). As shown in Figure 6E, a specific band of HMGB1 was detected only in STAT3 immunoprecipitated U266 lysate and not in normal rabbit IgG, suggesting an association of HMGB1 with STAT3 in MM cells.

### 3.6. Synergistic Activity of GS and BTZ in U266 Cells

**Figure 7:**
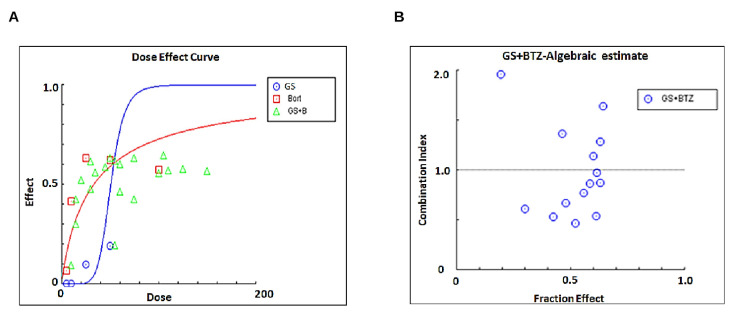
Calculating the synergistic concentrations required for a combination treatment with GS and BTZ. U266 cells were treated with 5, 10, 25, and 50 μM of GS or 5,10, 25, and 50 nM of BTZ alone or in different combinations to calculate the synergistic apoptotic response of GS and BTZ at 48 h. The concentration and fraction effect (**A**,**B**) graphs were created using CalcuSyn software. CI were calculated by employing Chou and Talalay’s methodology [41].

Next, we sought to investigate if subtoxic concentrations of GS and BTZ could potentiate the anticancer effects in U266 cells. BTZ is a food and drug administration (FDA)-approved proteasome inhibitor used to treat refractory MM patients [38]. However, the development of resistance [39] and relapse [40] have been shown to limit the efficacy of BTZ. To determine the combined concentrations of GS and BTZ showing maximal cytotoxic effects, U266 cells were treated with various concentrations of GS and BTZ combined, and the cell viability was determined. Using the Chou and Talalay method, the Combination Index (CI) of subtoxic concentrations of GS and BTZ was determined using CalcuSyn software [41,42]. According to this method, CI indicates the quantitative interaction among drugs, and CI values <1 indicate synergy, = 1 represents additive effect, and >1 shows antagonism. The synergistic effects of GS and BTZ on cell viability were observed at 10 µM of GS and 10 nM of BTZ concentrations with a CI index of 0.46825 (Figure 7A,B and Table 1).

### 3.7. GS sensitizes U266 Cells to BTZ

**Figure 8:**
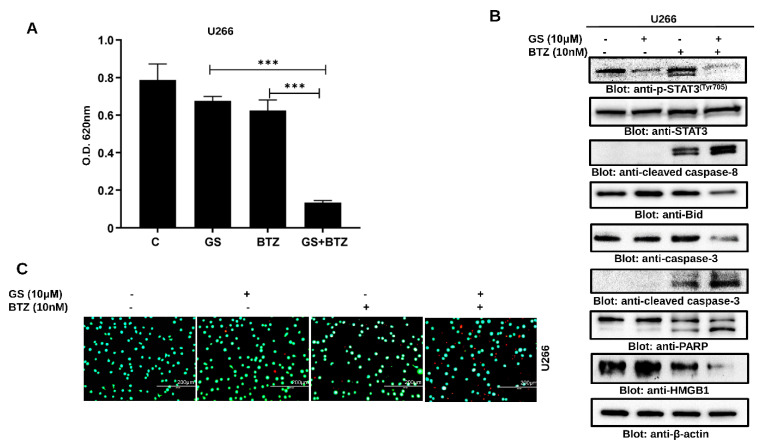
GS sensitizes MM cells to BTZ. U266 cells were incubated with 10 μM of GS and 10 nM of BTZ alone or in combination for 48 h. (**A**) The bar graph displays the cell viability determined by CCK-8 analysis. Data is presented as mean ± SD (n = 6. *** *p* < 0.0001). (**B**) The GS and BTZ- treated cells were lysed, and protein expressions of p-STAT3, STAT3, cleaved caspase-8, Bid, caspase-3, cleaved caspase-3, PARP, HMGB1, and β-actin were determined. (**C**) Cell viability using a live/dead cytotoxicity kit was also assessed (Scale bar, 200 μm). Full Western blot images can be found at Supplementary Materials File S1.

We further sought to determine whether GS can sensitize MM cells to BTZ when used in combination. We found that the combination of subtoxic concentrations of GS (10 µM) and BTZ (10 nM) significantly inhibited cell proliferation (Figure 8A) and induced cell death (Figure 8B) in U266 cells at 48 h compared to individual treatments of GS and BTZ. This apoptotic effect was attributed to a reduction in p-STAT3, Bid, and HMGB1 while induction in the activation of caspase-8, caspase-3, and cleavage of PARP (Figure 8B). The induction of cell death in the combination treatment was also evident in the increase in the number of non-viable cells (Figure 8C). Similar results were obtained in RPMI 8226 cells co-treatment with GS and BTZ potentiated anticancer effects as compared to either GS or BTZ alone treatment indicating a synergistic effect (Supplementary Figure S4).

## 4. Discussion

Recent advances in the treatment of MM have led to an unprecedented response rate and patient survival. Despite these improvements, the cure for MM remains elusive, highlighting the importance of discovering novel therapeutic targets and potent anticancer agents against MM [43]. GS, a phytosteroid of the plant *C. mukul*, possesses anticancer, anti-inflammatory, and antioxidant properties [28,44]. Hence, in this study, we sought to evaluate the anticancer potential of GS in MM cells. Our findings demonstrate that GS exerts a concentration-dependent anti-proliferative effect on MM cells and induces cell death by activating extrinsic and intrinsic apoptotic pathways. These findings agree with previous studies that have documented GS-mediated apoptosis in breast cancer, ovarian cancer, and hepatocellular carcinoma [27,45]. Moreover, the caspase-dependent apoptotic effect of GS was confirmed using a pan-caspase inhibitor, z-VAD-FMK, which prevented GS-mediated apoptosis. This further confirmed that GS induced apoptosis in MM cells through caspase cascade activation.

HMGB1 is actively involved in tumor growth, cell proliferation, metastasis, and invasion [46]. This makes HMGB1 a critical target in cancer therapy. In addition to HMGB1, the dysregulation of the JAK/STAT pathway causes resistance to apoptosis in multiple human malignancies [47,48]. Therefore, the role of HMGB1 in MM cells and its association with the JAK/STAT signaling pathway were elucidated. GS-mediated anticancer activity involves the downregulation of HMGB1 in MM cells. Furthermore, the inhibition of JAK-2 and STAT3 using specific inhibitors, such as AG-490 and static and the siRNA knockdown of STAT3 resulted in the downregulation of HMGB1 protein expression. Previous studies have shown that suppression of STAT3 using specific inhibitors suppresses the growth of cancer cells [49]. AG-490 is reported to significantly decrease the expression of p-STAT3 at a concentration of 50 µM [49]. Interestingly, our results demonstrate the association of the JAK/STAT signaling pathway with HMGB1 and that HMGB1 expression depends on the activation of the JAK/STAT signaling pathway.

The activation of STAT3 regulates the transcription of various antiapoptotic genes, such as Bcl-xL. Our results demonstrated that GS suppressed the phosphorylation of STAT3, thereby downregulating the downstream targets of STAT3, including Bcl-xL, cyclin D, and XIAP. In addition to these molecular targets, the expression of a PTP, SHP-1, was upregulated upon GS treatment which was associated with the downregulation of STAT3 phosphorylation. PTPs are negative regulators of the JAK/STAT signaling pathway [50], and SHP-1 regulates the STAT3 activation. Similar observations were made by Ahn and the group, who reported SHP-1 upregulation in MM cells upon GS treatment [29].

The involvement of IL-6 in MM pathogenesis and inhibition of IL-6-mediated STAT3 activation by other anticancer agents in MM cells has been described earlier [48,51]. Our results align with these studies as GS also inhibited the secretion of IL-6 and downregulated the IL-6-mediated activation of STAT3 in U266 and RPMI 8226 cells. Interestingly, treatment of MM cells with GS in the presence of IL-6 resulted in the suppression of p-STAT3 and downregulation of HMGB1. Therefore, we conclude that GS inhibits IL-6 secretion that eventually suppresses the activation of STAT3, thereby downregulating the expression of HMGB1 and inhibiting MM cell proliferation. This confirmed that HMGB1 expression is associated with STAT3 in MM cells, suggesting a role of the JAK/STAT3/HMGB1 axis in the pathogenesis of MM.

In recent years, combination therapy involving two or more therapeutic agents to enhance the efficacy of anticancer drugs has gained widespread attention. Although the conventional monotherapy approach is still a standard treatment modality for different types of cancer, it is less effective than the combination therapy approach as it is more vulnerable to drug resistance. Usually, it targets proliferating cells non-selectively, leading to the death of both healthy and tumor cells [52]. Combination therapy involving two or more drugs that exhibit potent anticancer effects can prevent the cytotoxic effects on healthy cells while simultaneously killing the tumor cells. This therapy usually works synergistically or in an additive form, requiring a low therapeutic dosage of each drug. In our study, we observed that the combination of GS and BTZ significantly reduced the cell viability of U266 cells synergistically. Moreover, we observed that GS sensitized U266 cells to BTZ, inhibiting proliferation and inducing cell death when used in combination. Our results are consistent with those of earlier studies in which GS was reported to increase the anticancer effects of chemotherapeutic drugs, such as cisplatin, cetuximab, erlotinib, and bortezomib in HNSCC [53,54] and gemcitabine in pancreatic cancer [29]. Thus, a thorough evaluation of the impact and therapeutic potential of combining GS with other anticancer drugs in MM is still required.

**Figure 9:**
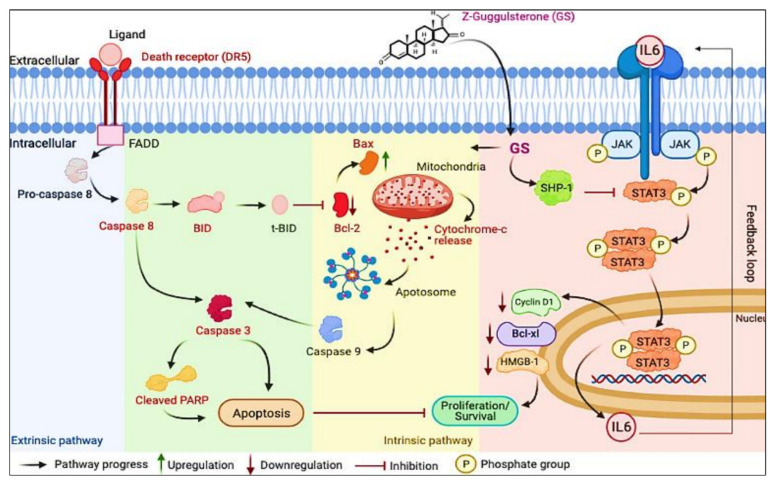
Schematic diagram representing a model for GS-induced cytotoxicity in MM cells plausibly through the suppression of the JAK/STAT signaling pathway and induction of apoptosis.

In the current study, we explored the anticancer effect of GS treatment on MM cells by evaluating the intrinsic and extrinsic apoptotic pathways. The findings of our study are consistent with other studies, where GS induced anticancer effects in gastric cancer cells and cholangiocarcinoma and promoted apoptosis via the intrinsic pathway [44,55]. Our study also documented, for the first time, the cytotoxic effects of GS by inactivating the JAK/STAT signaling activity and the downregulation of HMGB1 (summarized in Figure 9). We also showed that GS upregulates the expression of SHP-1 and consequently inhibits the activation of STAT3.

Despite the fact that the currently available drugs have, to some extent, significantly improved the quality of life of MM patients, the clinical outcome is still unsatisfactory due to toxicity, relapse, and acquired drug resistance, which has emerged as the primary barrier to chemotherapy’s failure in the clinical treatment of MM. Therefore, further exploration of the potential alternatives to existing therapeutic options and their molecular mechanisms is highly urgent for seeking effective treatment strategies for MM. The expression of HMGB1 is upregulated in various tumor tissues compared to normal tissues. This upregulation is associated with tumor invasion, metastasis, progression, and other aspects of tumor growth. Additionally, HMGB1 can be produced from a variety of tumor cells in response to chemo or radiation therapy, and as a result, it plays a role in the regulation of cancer cells’ chemoresistance and sensitivity. The proteasome inhibitor BTZ, has marked efficacy against MM, but the major limitations of this drug are intrinsic and acquired resistance. Considering all these factors, we thought of investigating the potential of GS as a natural anticancer drug alone and in combination with BTZ in MM cells. The results of this study, which show that GS can target STAT3 and HMGB1 and improve the effectiveness of BTZ, suggest the therapeutic relevance of this safe and affordable nutraceutical as a viable supplemental medicine for the treatment of MM. Understanding the molecular underpinnings of HMGB1′s role as a protumor protein in cancer requires additional research. Additionally, even though numerous xenografts or transplantable tumor models have been employed, generating HMGB1-associated spontaneous tumor models is crucial.

In conclusion, our results elucidated that GS, as a single agent, is active against MM. However, the toxicity and bioavailability studies of GS have not been performed yet in cancer clinical studies. Therefore, this is an area of future investigation. Our study offers proof of concept for more research into the function of HMGB1 and the possible clinical use of GS in treating MM. It also provides a preclinical framework for integrating GS and BTZ in clinical settings. Our study, along with other previously published studies, provides synthetic and structural biologists with some justification to modulate the chemistry of this alkaloid, such as active moiety, including its phenolic and alcoholic groups, the unsaturation pattern, and the presence of an amine group, to make this compound more therapeutically feasible with increased bioavailability and less toxicity.

## Figures and Tables

**Table 1:** Combination Index calculation using Chou and Talalay method in U266 cell line.

GS (µM)	Bortezomib (nM)	Fractional Effect (Fa)	Combination Index (CI)	Concentration Reduction Index (DRI) GS (µM)	Concentration Reduction Index (DRI) Bortezomib (nM)
5	5	0.096	2.04474	7.404	0.523
10	10	0.524	0.46825	5.115	3.666
25	25	0.633	0.87011	2.177	2.434
50	50	0.556	2.13938	1.041	0.848

## Data Availability

As per MDPI Research Data Policies.

## Supplementary Materials

The following supporting information can be downloaded at: https://www.mdpi.com/article/10.3390/cancers14225621/s1, File S1: Full Western blot images. Figure S1: Anti-proliferative effects of GS on MM cells. GS inhibits the viability of (**A**) MM.1S (**B**) RPMI 8226 in a concentration-dependent manner after treatment with 5, 10, 25, and 50 µM of GS for 48 h. The graphs display the mean ± SD of three independent experiments with replicates. * *p* < 0.05, ** *p* < 0.001. Figure S2: IC50 values were calculated after treatment with 5, 10, 25, and 50 µM GS for (**A**–**C**) U266, (**D**–**F**) RPMI 8226 and (**G** and **H**) MM.1S cells. Figure S3: (**A**) Graphical representation of live and dead analysis for Figure 1D. The graph displays mean ± SD where n = 3 and ** *p* <0.001. (**B**) The viability of RPMI 8226 cells was evaluated using a live/dead cytotoxicity kit at GS concentrations of 5, 10, 25, and 50 µM. Green denotes live cells, while red denotes non-viable cells (Scale bar, 200 μm). (**C**) Involvement of PTPs in GS-induced downregulation of STAT3. Total proteins were extracted from U266 cells, resolved using SDS-PAGE, and probed against SHP-1 and HSP60. Figure S4: GS sensitizes MM cells to BTZ. RPMI8226 cells were incubated with 10 μM of GS and 5 nM of BTZ alone or in combination for 24 h. (**A**) The bar graph displays the cell viability determined by CCK-8 analysis. Data is presented as mean ± SD (n = 6. ******* *p* < 0.0001). (**B**) The GS and BTZ- treated cells were lysed, and protein expression of Casapas-3, cleaved caspase-3. cleaved caspase-8, and GAPDH was determined. (**C**) Cell viability using a live/dead cytotoxicity kit was also assessed (Scale bar, 200 μm).
