# Supplementary material for: Guggulsterone Induces Apoptosis in Multiple Myeloma Cells by Targeting High Mobility Group Box 1 via Janus Activated Kinase/Signal Transducer and Activator of Transcription Pathway
Source: Cancers (Basel). 2022 Nov 16;14(22):5621. doi: 10.3390/cancers14225621 (PMC9688888; doi:10.3390/cancers14225621)

Figure S3

U266

Figure S3D

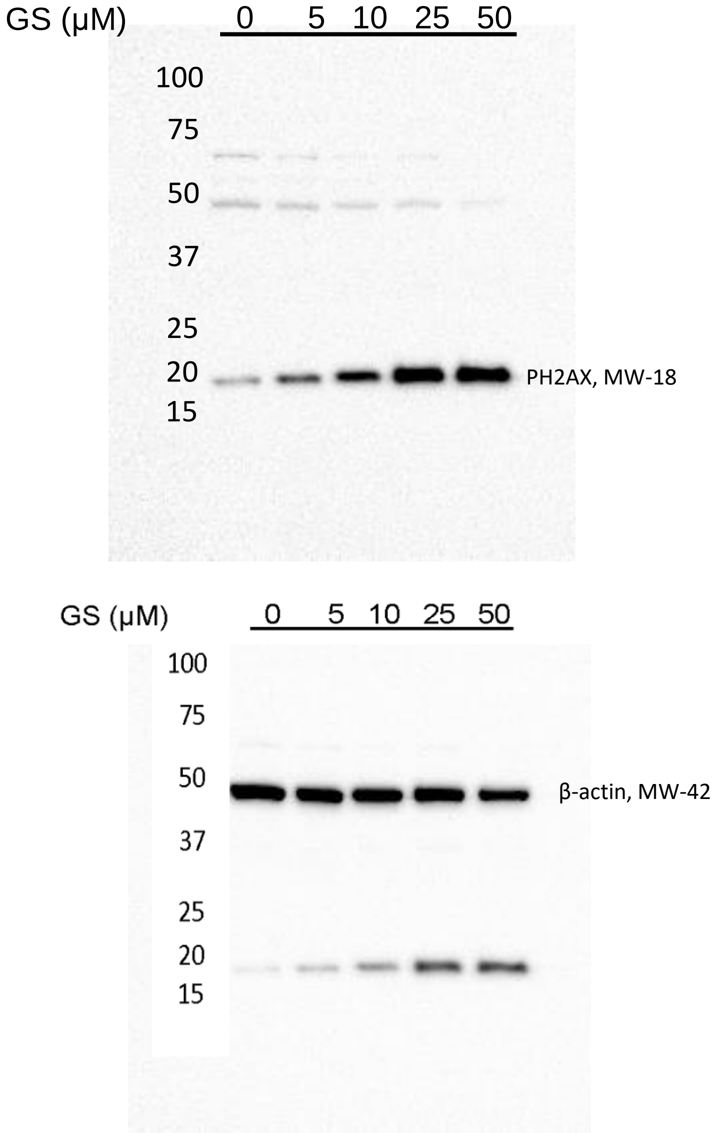

RPMI 8226

Figure S3E

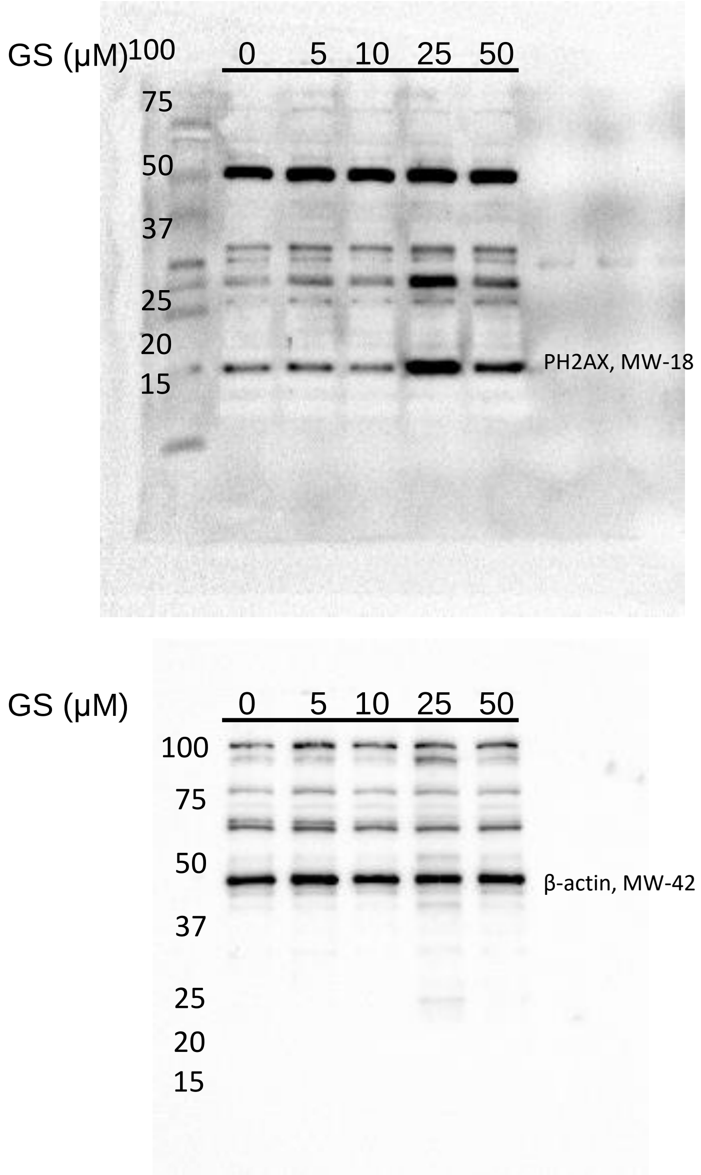

Figure 4

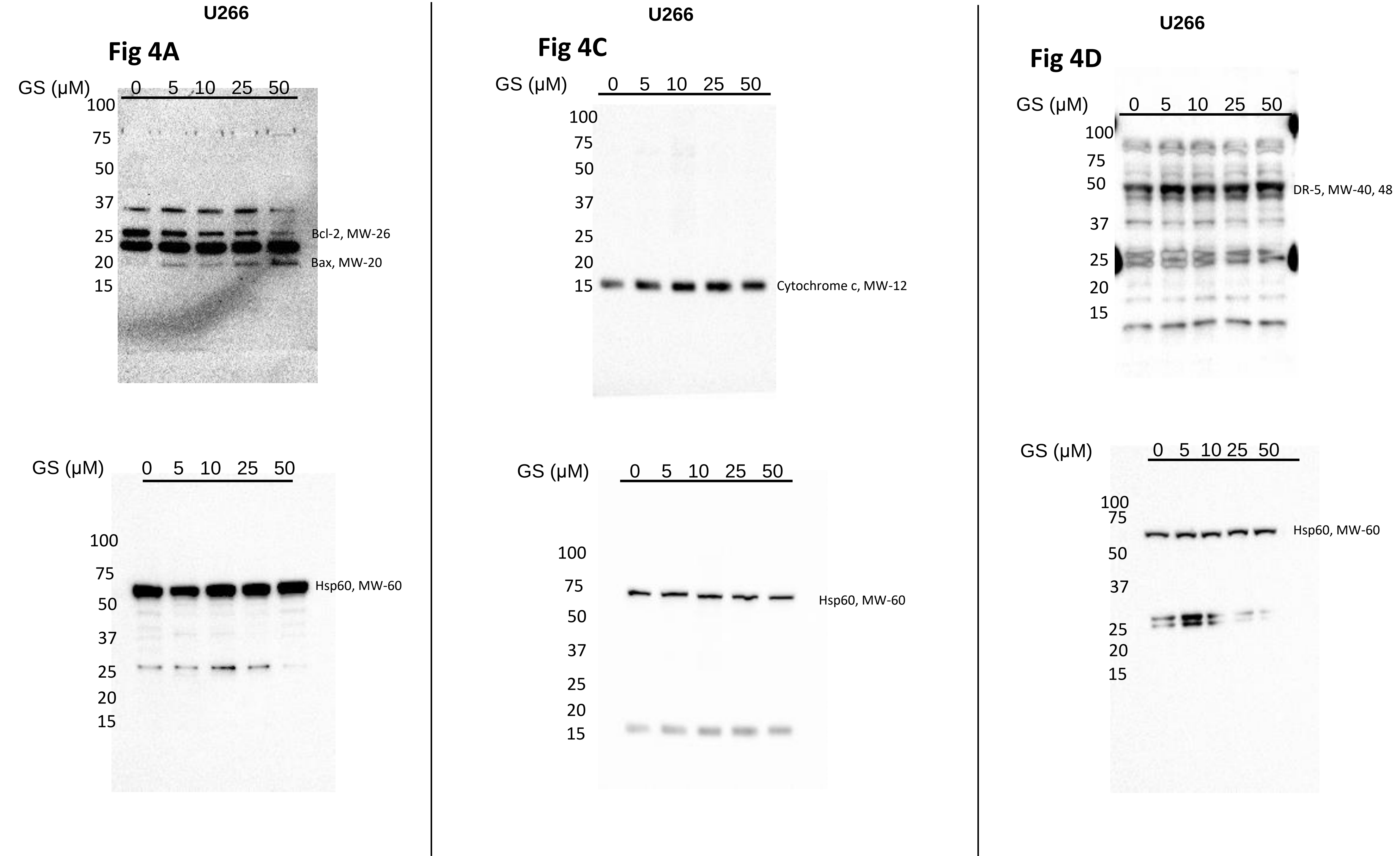
U266
Fig 4A
GS (µM) 0 5 10 25 50
100
75
50
37
25
20
15
Bcl-2, MW-26
Bax, MW-20
GS (µM) 0 5 10 25 50
100
75
50
37
25
20
15
Hsp60, MW-60
U266
Fig 4C
GS (µM) 0 5 10 25 50
100
75
50
37
25
20
15
Cytochrome c, MW-12
GS (µM) 0 5 10 25 50
100
75
50
37
25
20
15
Hsp60, MW-60
U266
Fig 4D
GS (µM) 0 5 10 25 50
100
75
50
37
25
20
15
DR-5, MW-40, 48
GS (µM) 0 5 10 25 50
100
75
50
37
25
20
15
Hsp60, MW-60

Figure 4

U266

Fig 4D

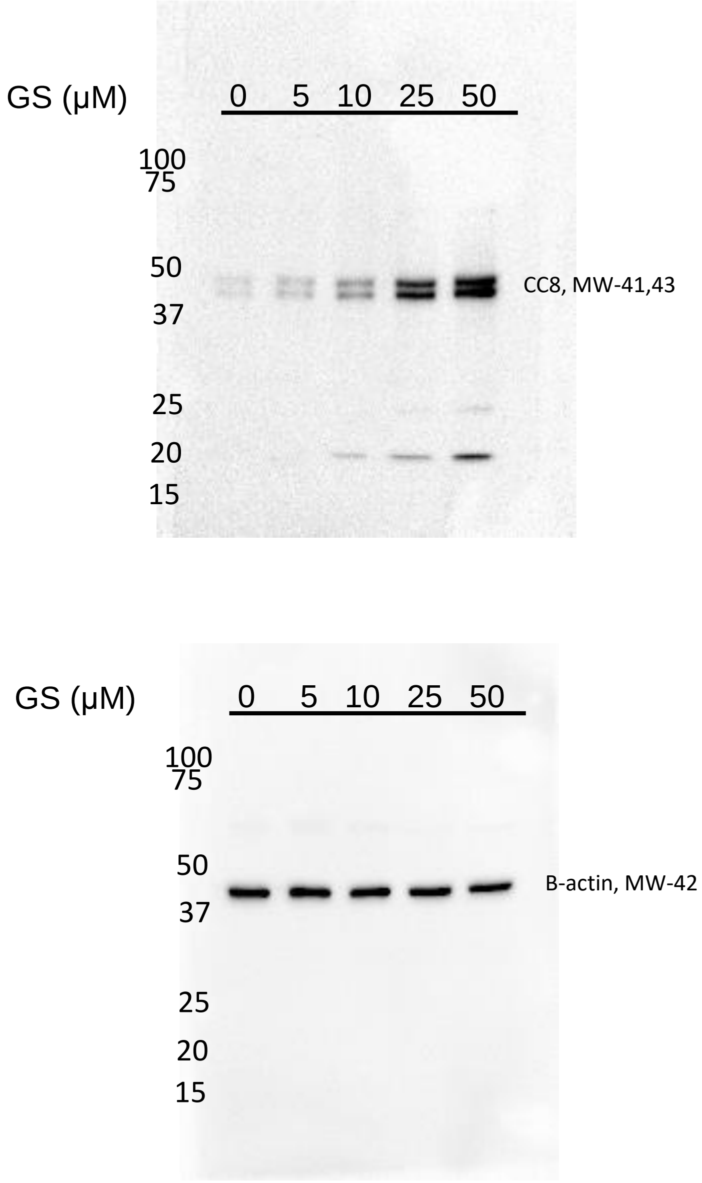

RPMI 8226

Fig 4E

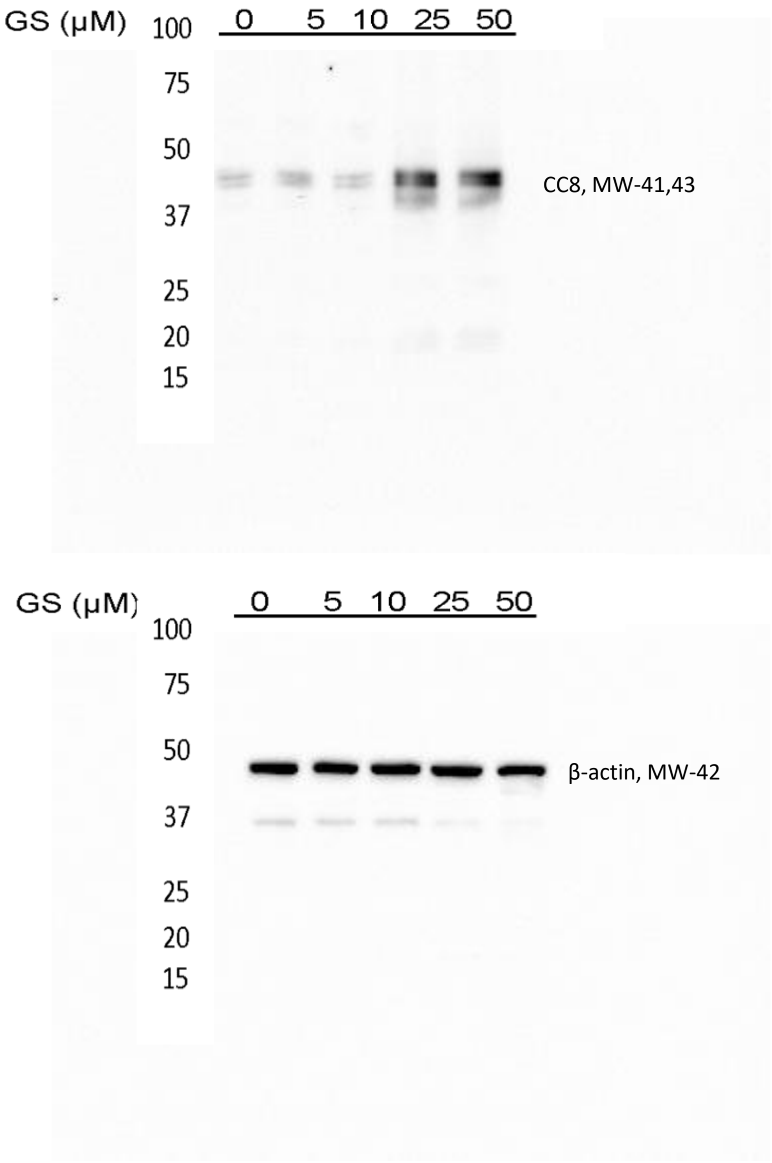

Figure 5

U266

Fig 5A

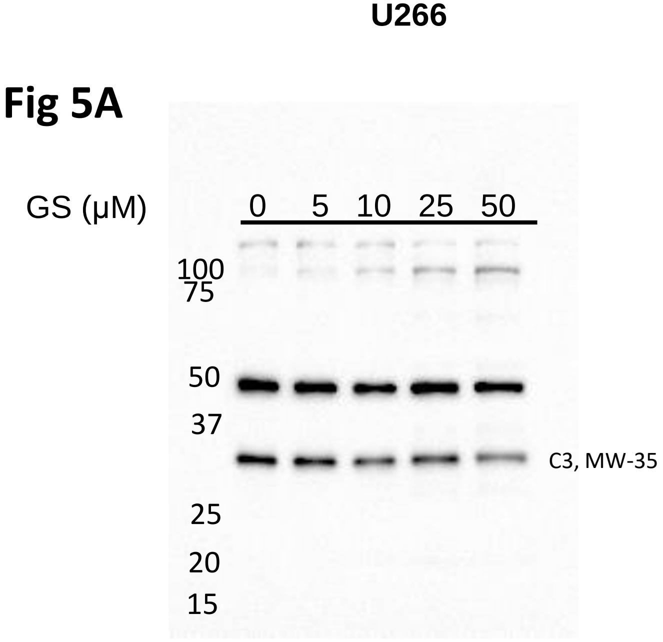

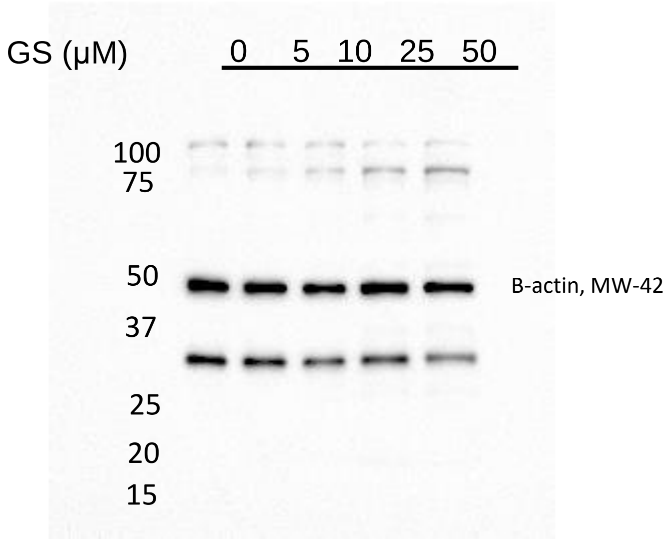

U266

Fig 5A

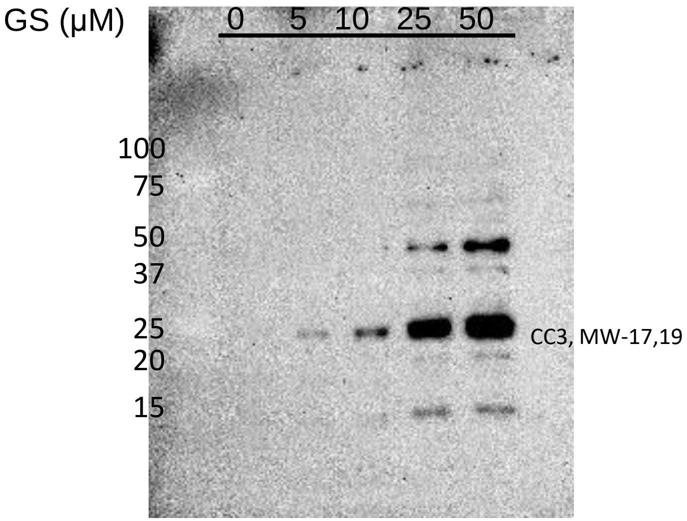

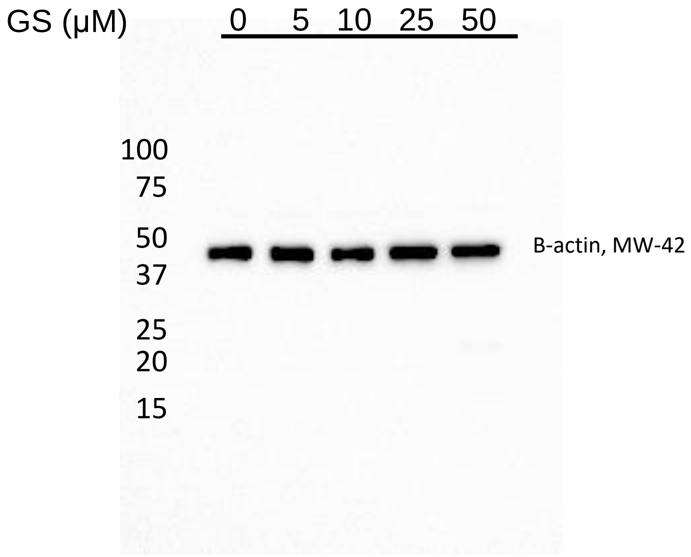

U266

Fig 5A

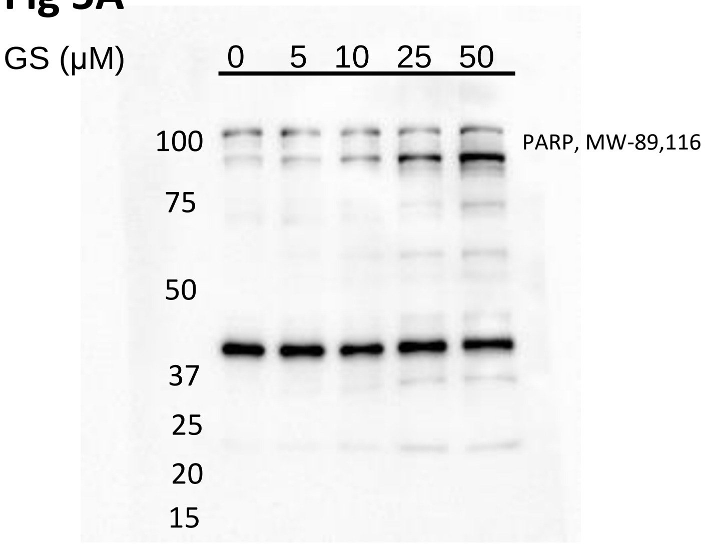

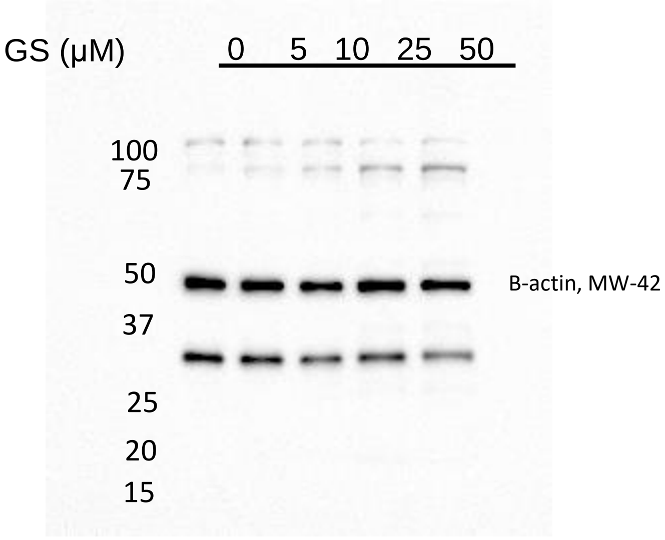

Figure 5

RPMI 8226

Fig 5B

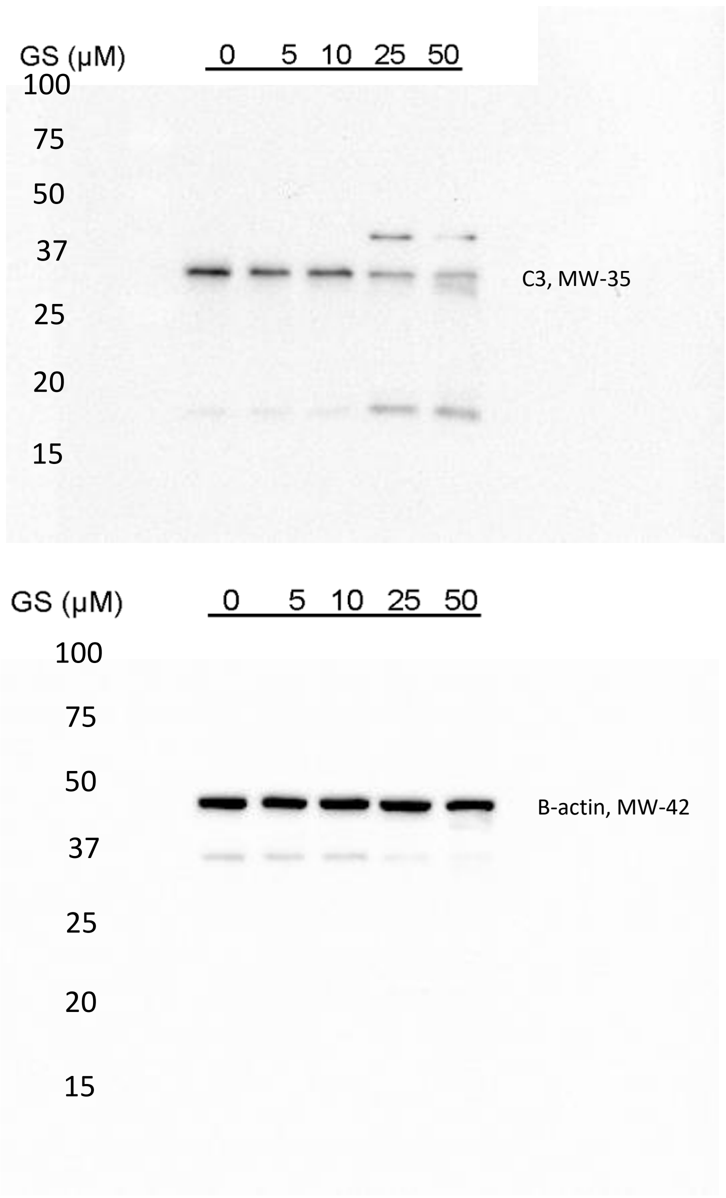

RPMI 8226

Fig 5B

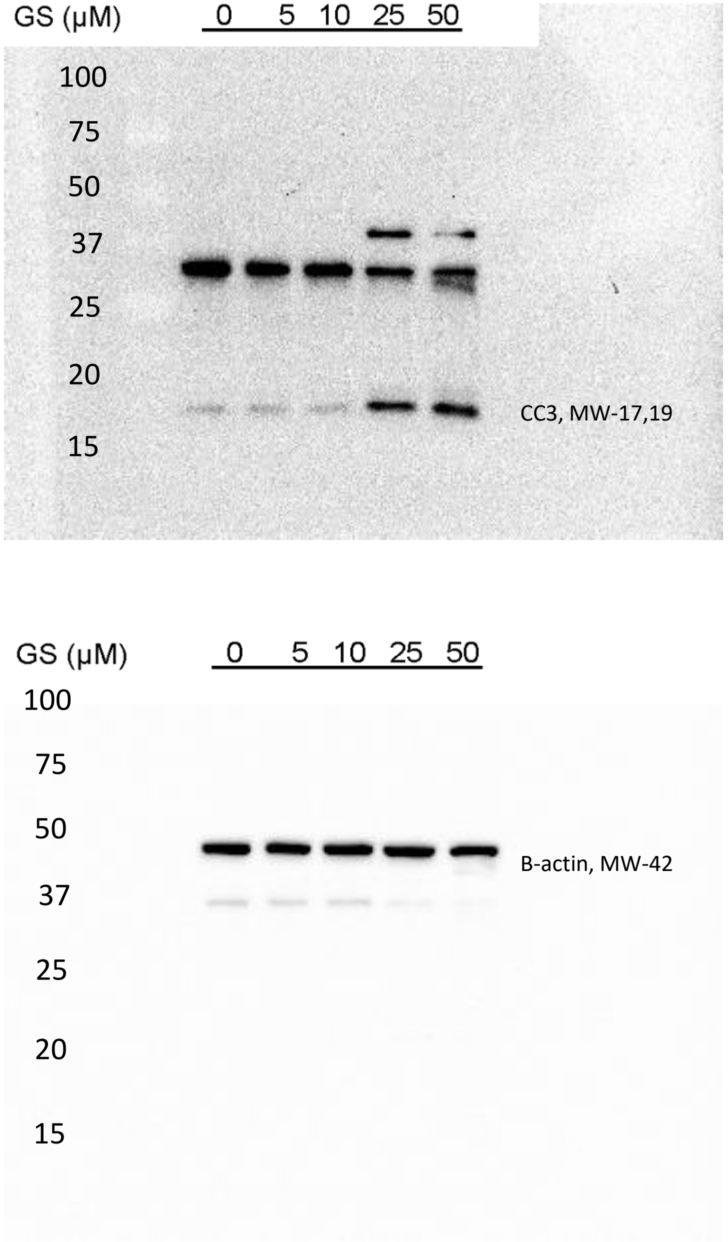

RPMI 8226

Fig 5B

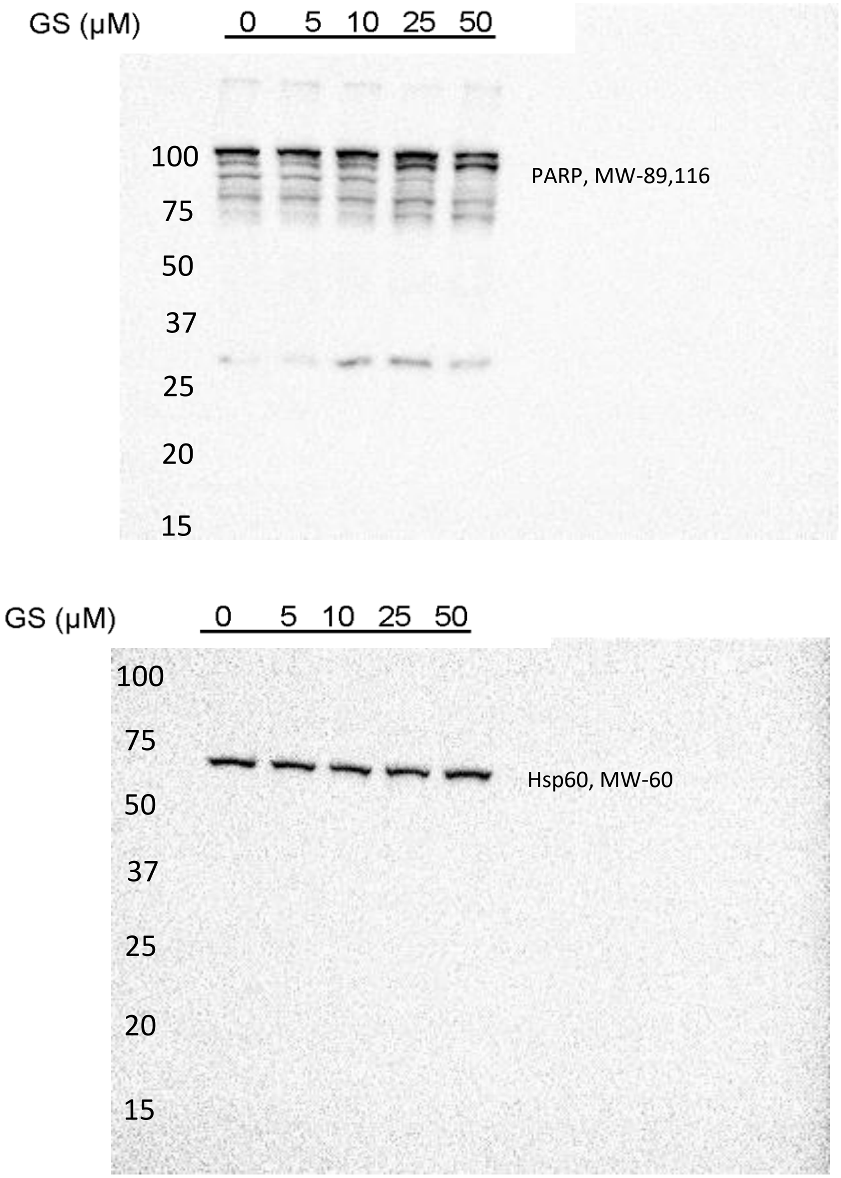

Figure 5

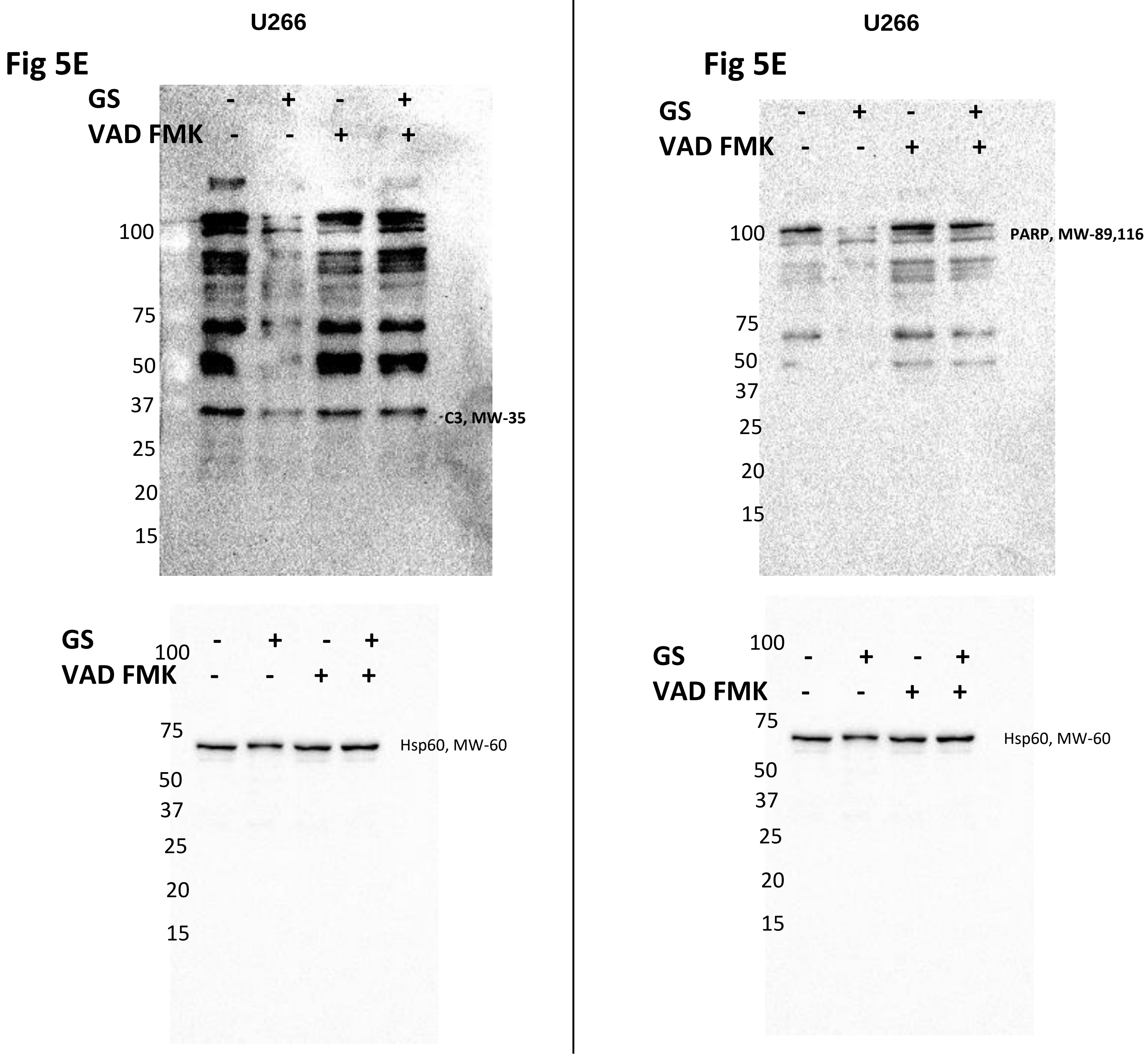
U266
Fig 5E
GS - + - +
VAD FMK - - + +
100
75
50
37
25
20
15
C3, MW-35
GS - + - +
VAD FMK - - + +
100
75
50
37
25
20
15
Hsp60, MW-60
U266
Fig 5E
GS - + - +
VAD FMK - - + +
100
75
50
37
25
20
15
PARP, MW-89,116
GS - + - +
VAD FMK - - + +
100
75
50
37
25
20
15
Hsp60, MW-60

Figure 6

U266

Fig 6B

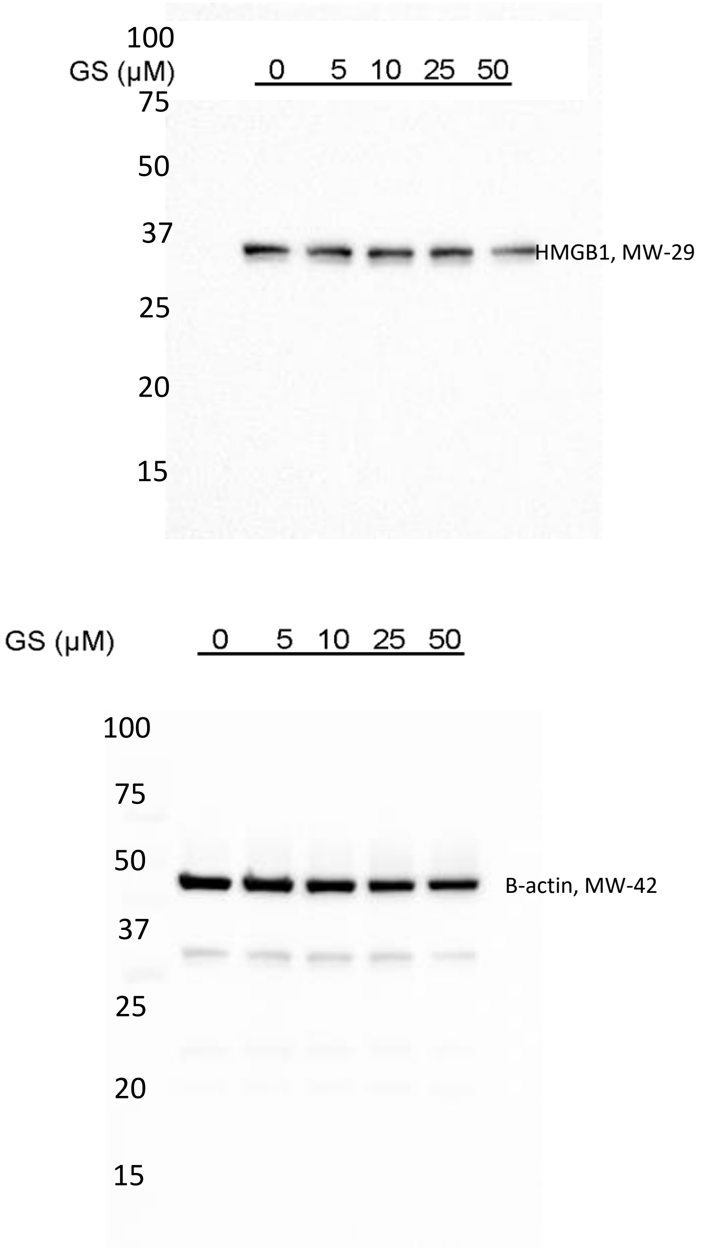

RPMI 8226

Fig 6C

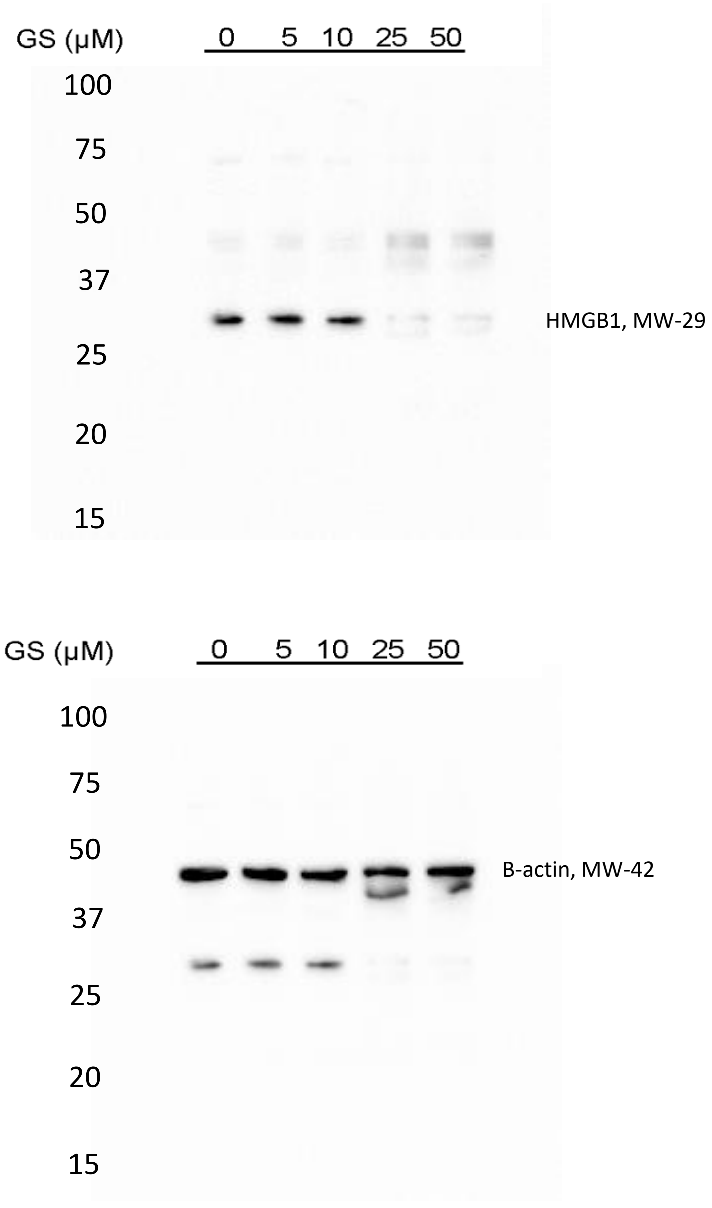

U266

Fig 6D

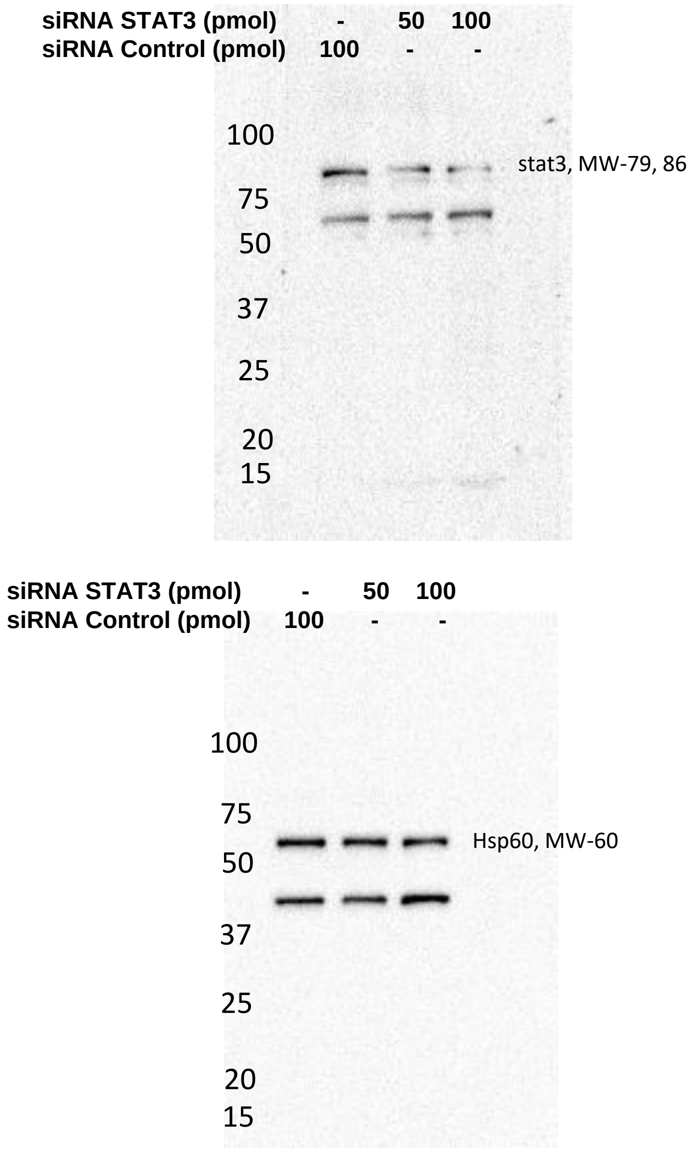

Figure 6

U266

Fig 6D

U266

Fig 6D

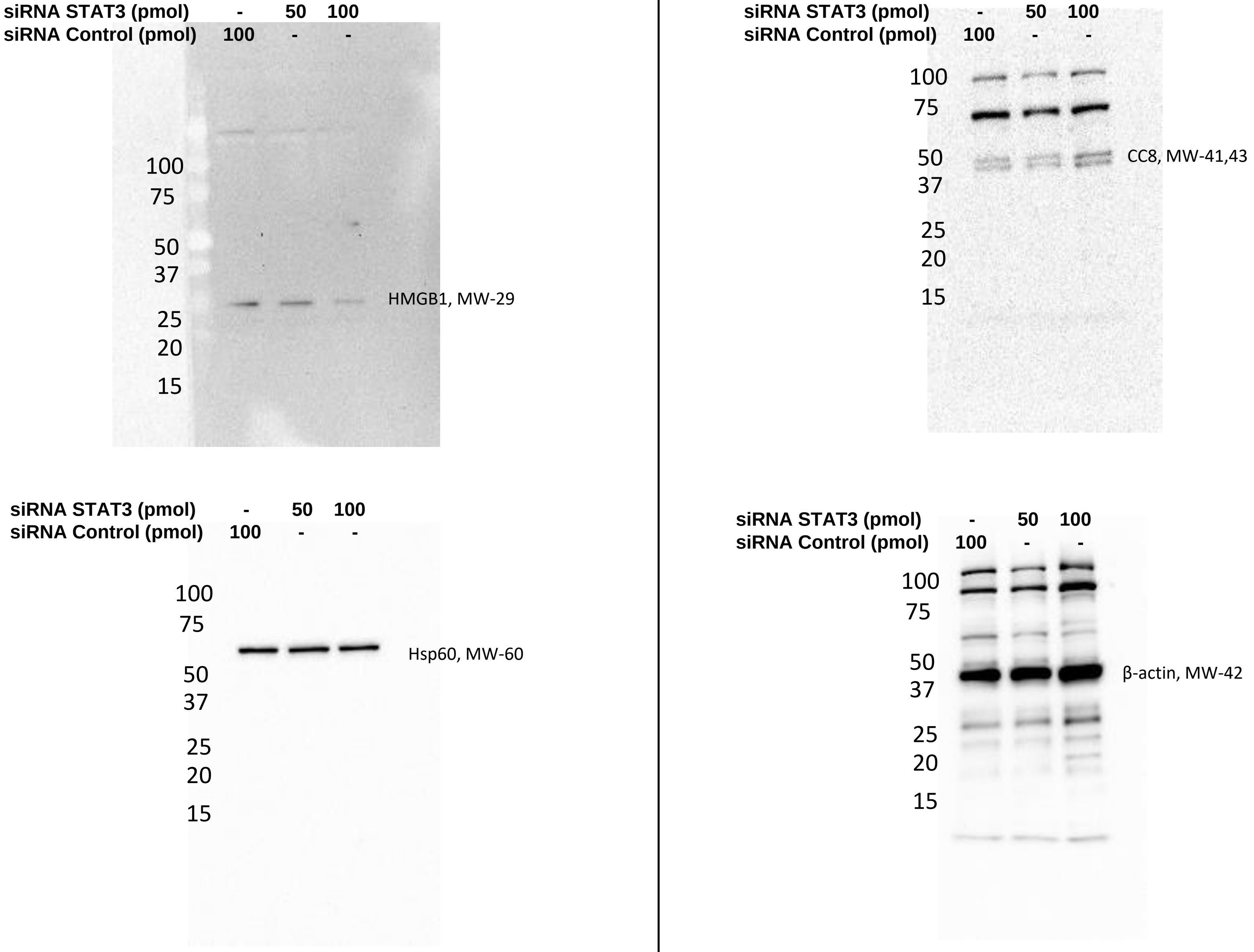

Figure 6

U266

Fig 6F

GS (50µM) - + - -
AG-490 (50µM) - - + -
Stattic (10µM) - - - +

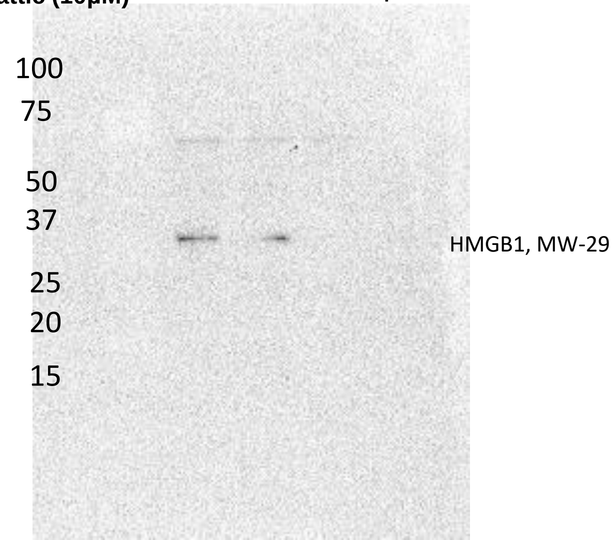

GS (50µM) - + - -
AG-490 (50µM) - - + -
Stattic (10µM) - - - +

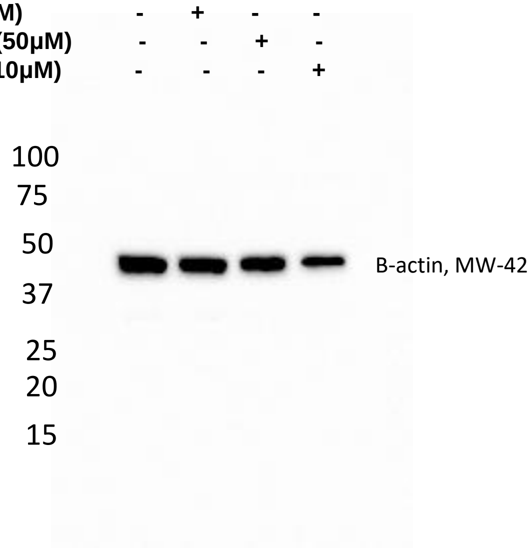

U266

Fig 6F

GS (50µM) - + - -
AG-490 (50µM) - - + -
Stattic (10µM) - - - +

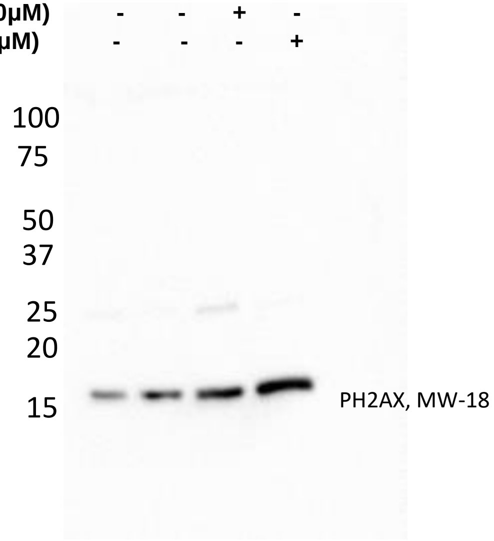

GS (50µM) - + - -
AG-490 (50µM) - - + -
Stattic (10µM) - - - +

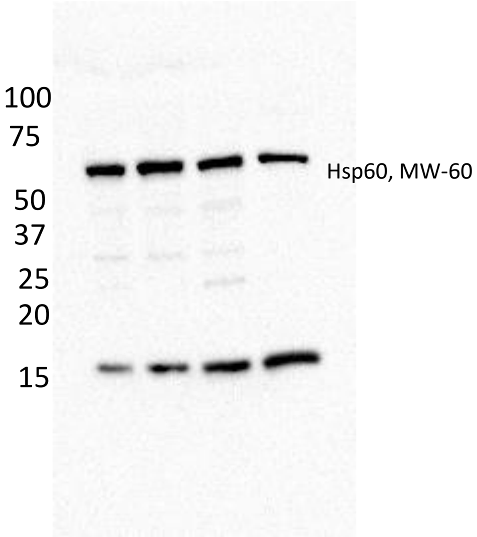

Figure 7

U266

Fig 7A

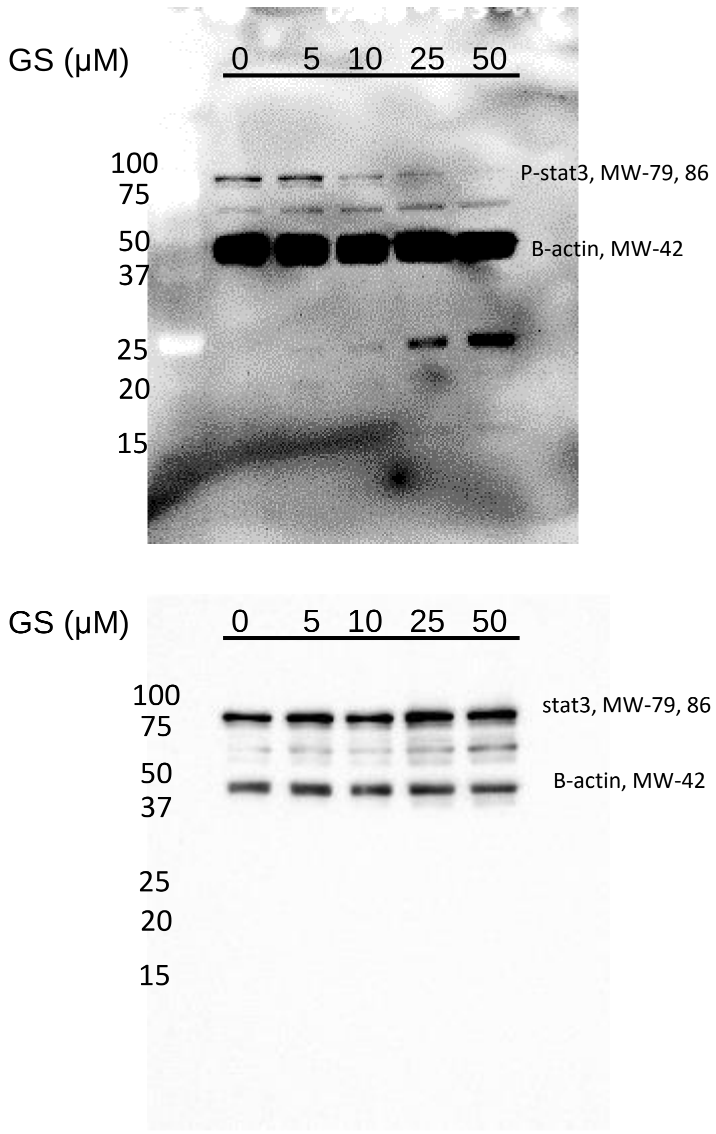

U266

Fig 7A

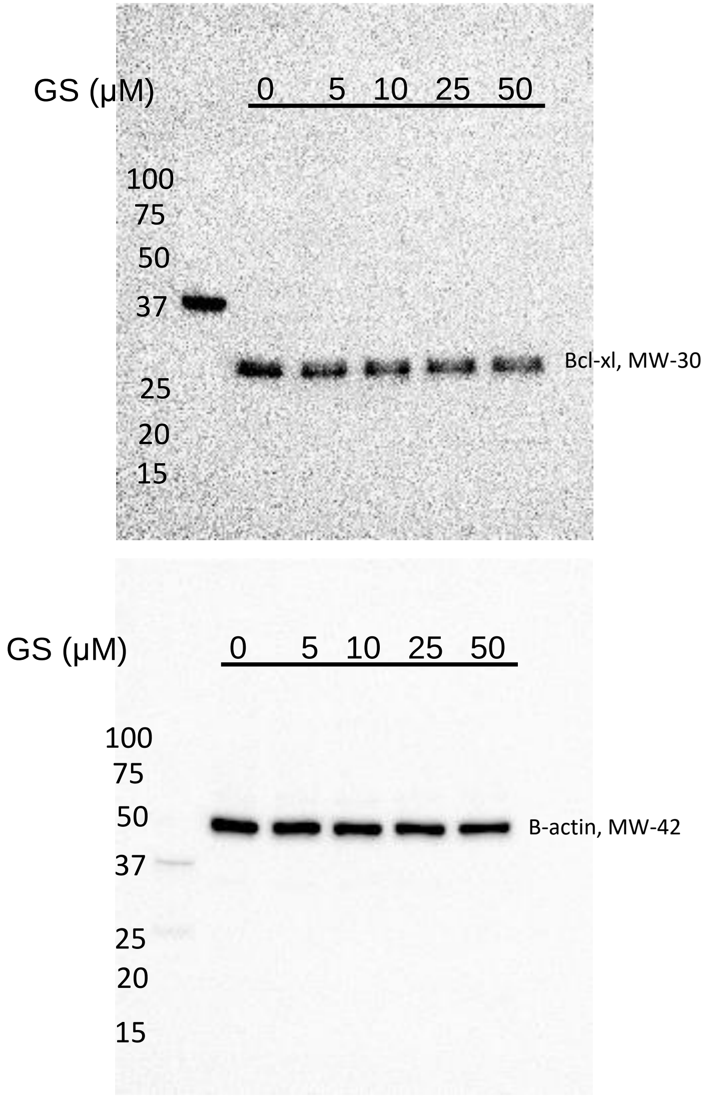

U266

Fig 7A

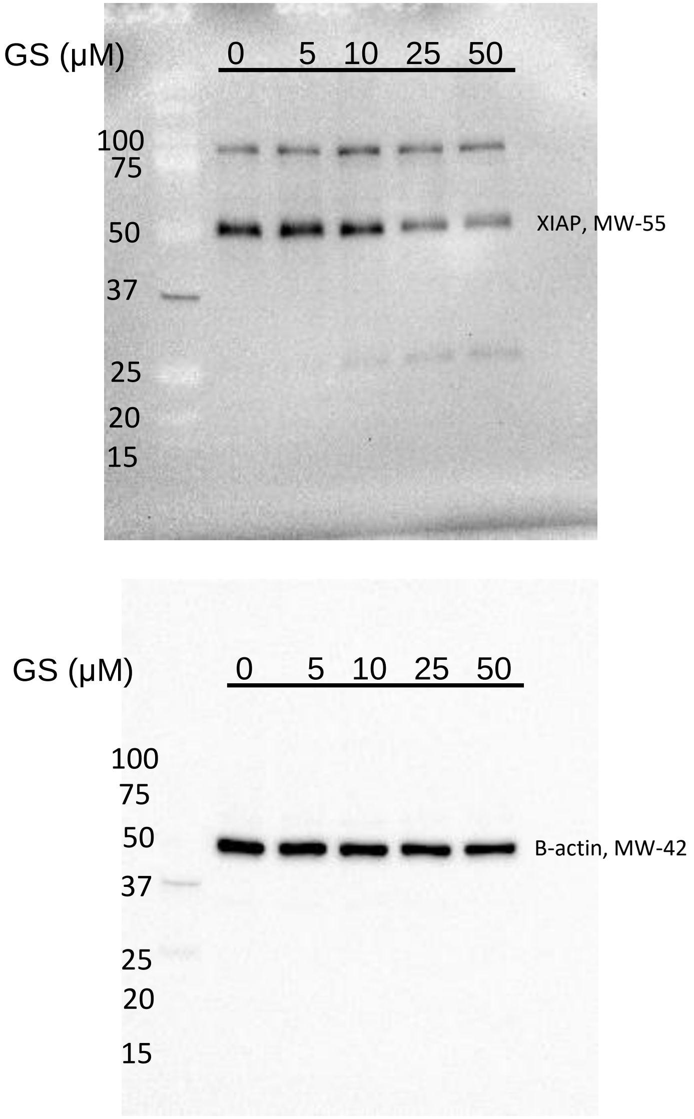

Figure 7

U266

Fig 7A

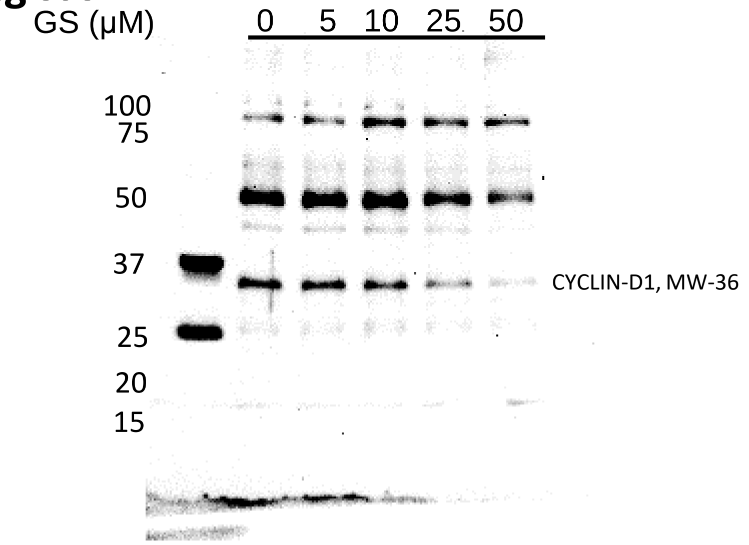

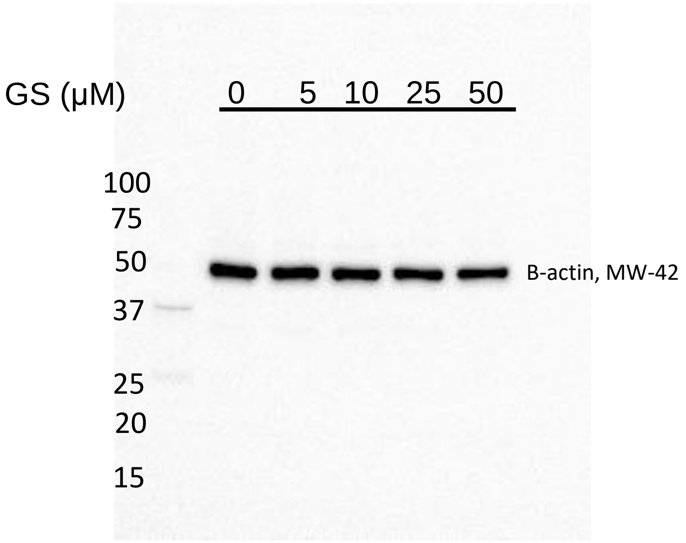

U266

Fig 7A

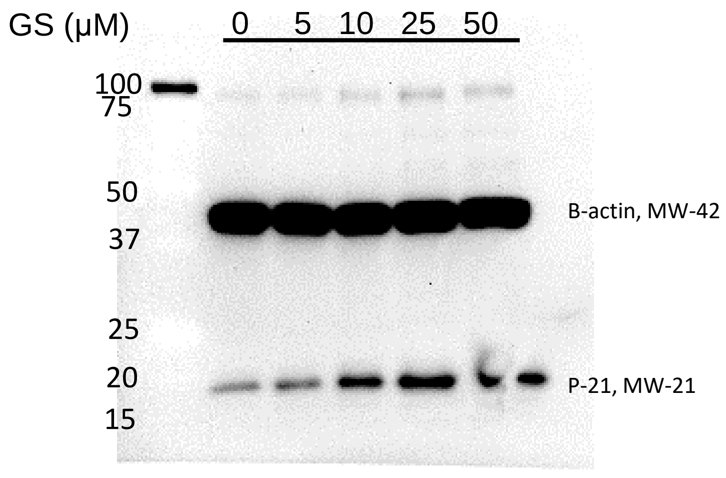

Fig 7A

U266

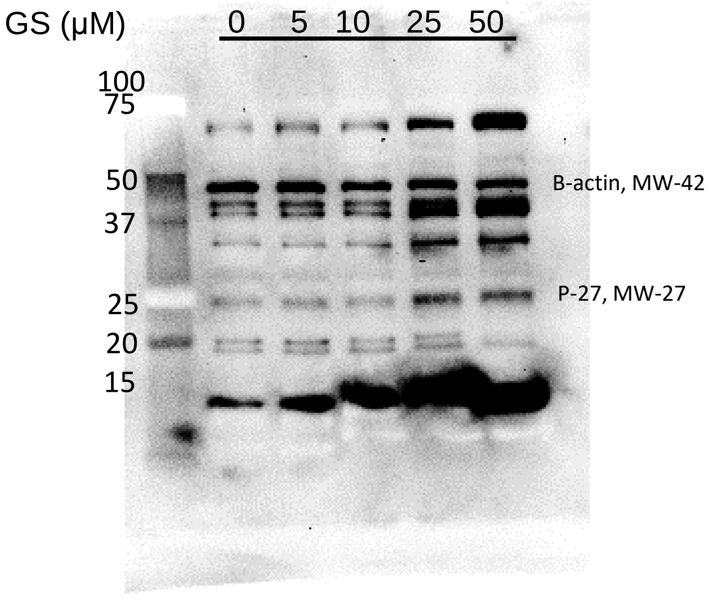

Figure 7

U266

Fig 7C

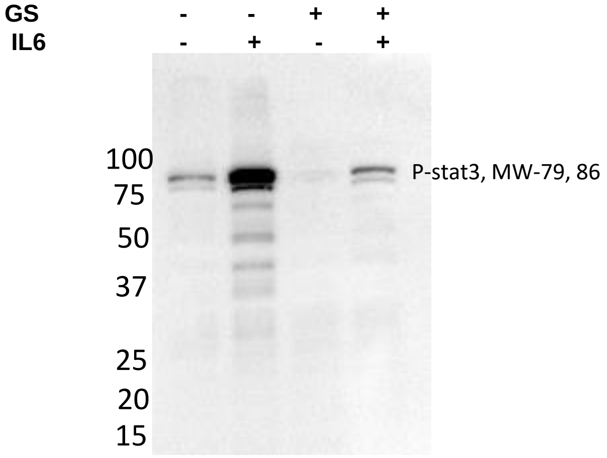

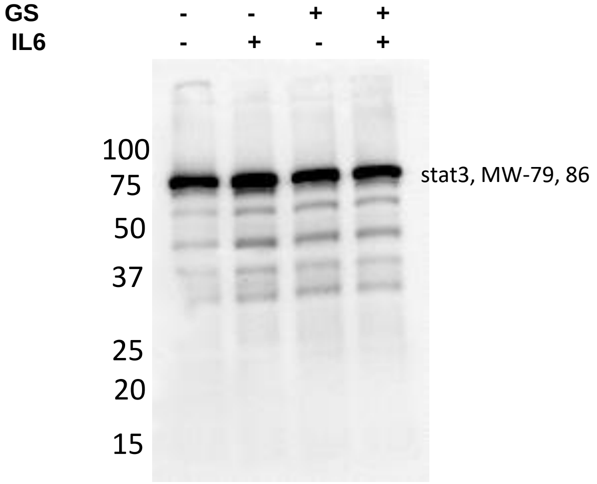

U266

Fig 7C

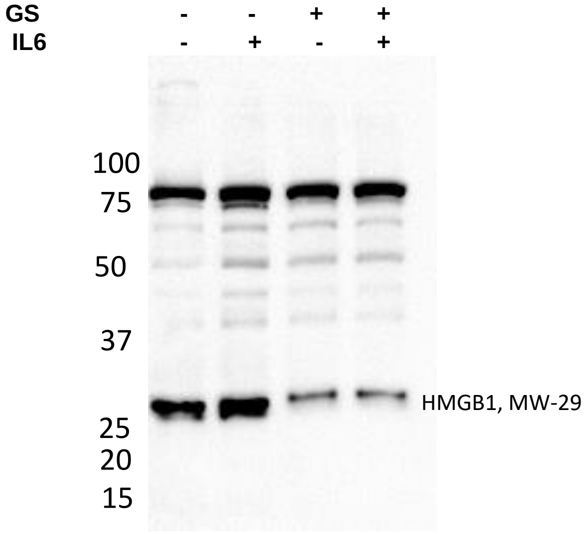

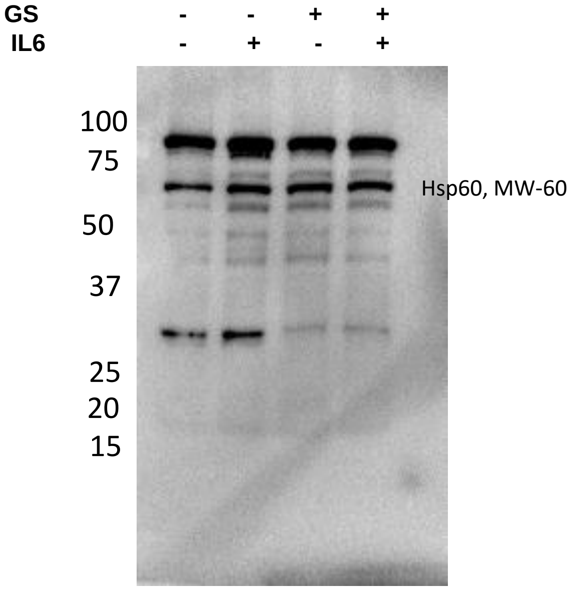

Figure 7

RPMI 8226

Fig 7D

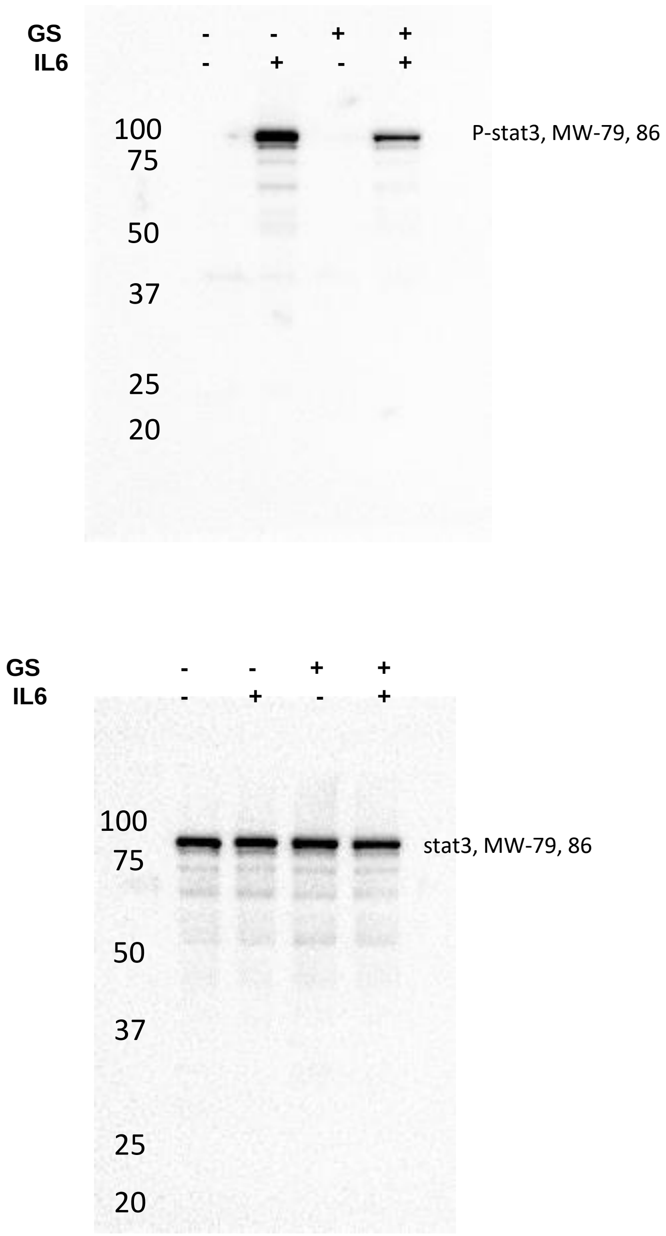

U266

Fig 7E

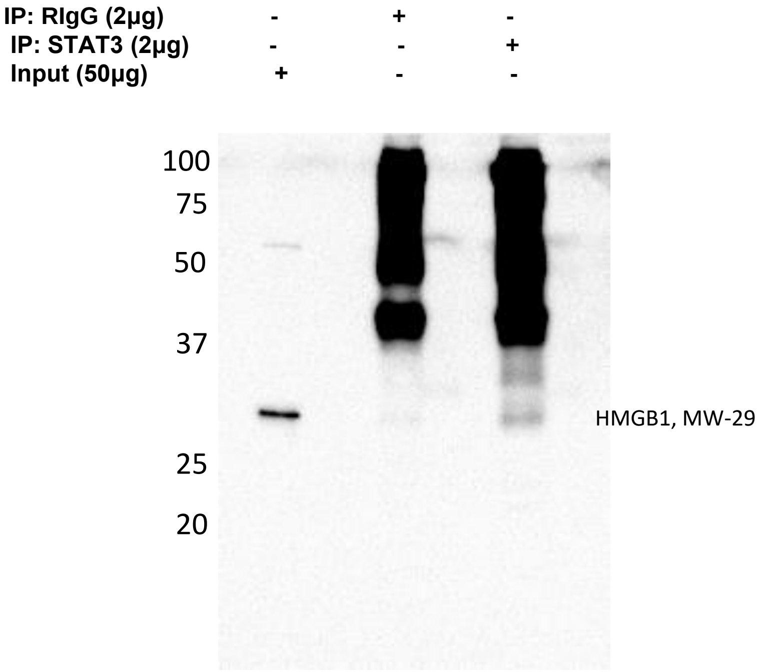

Figure 9

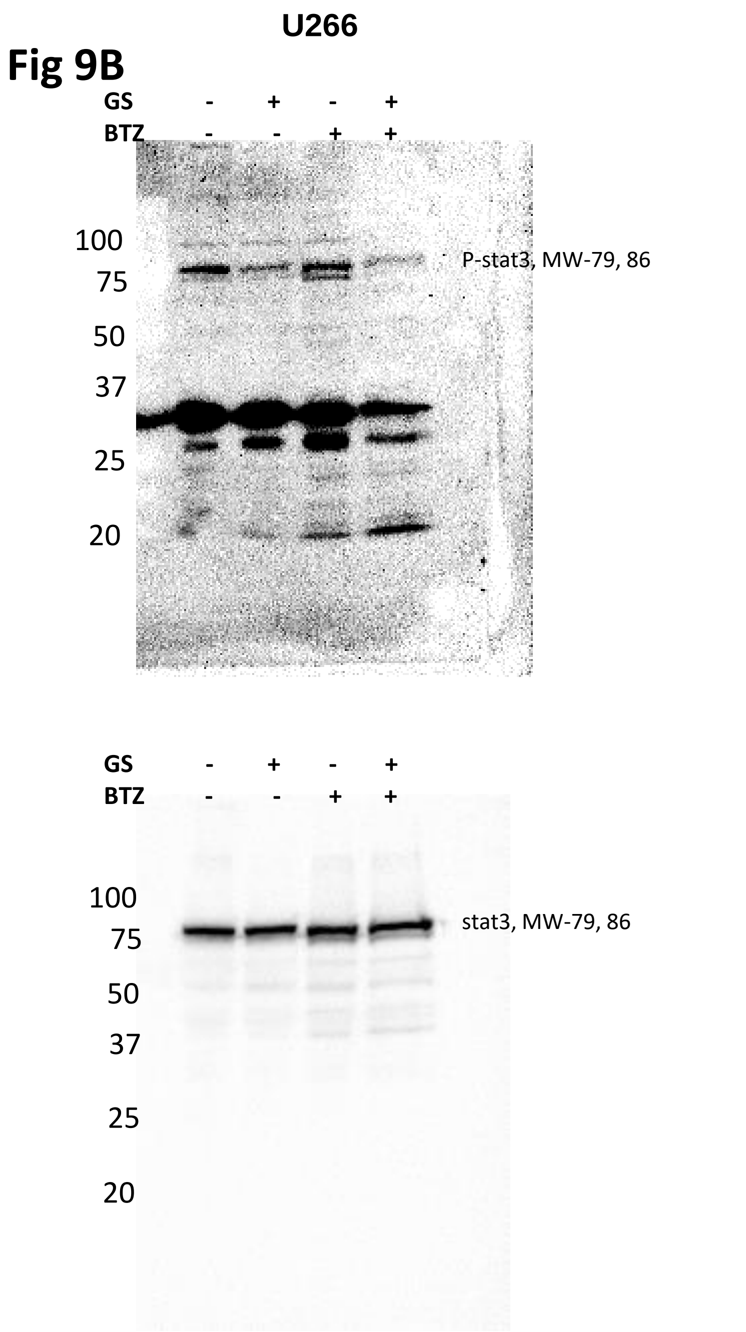

U266
Fig 9B
GS - + - +
BTZ - - + +
100
75
50
37
25
20
P-stat3, MW-79, 86
GS - + - +
BTZ - - + +
100
75
50
37
25
20
stat3, MW-79, 86

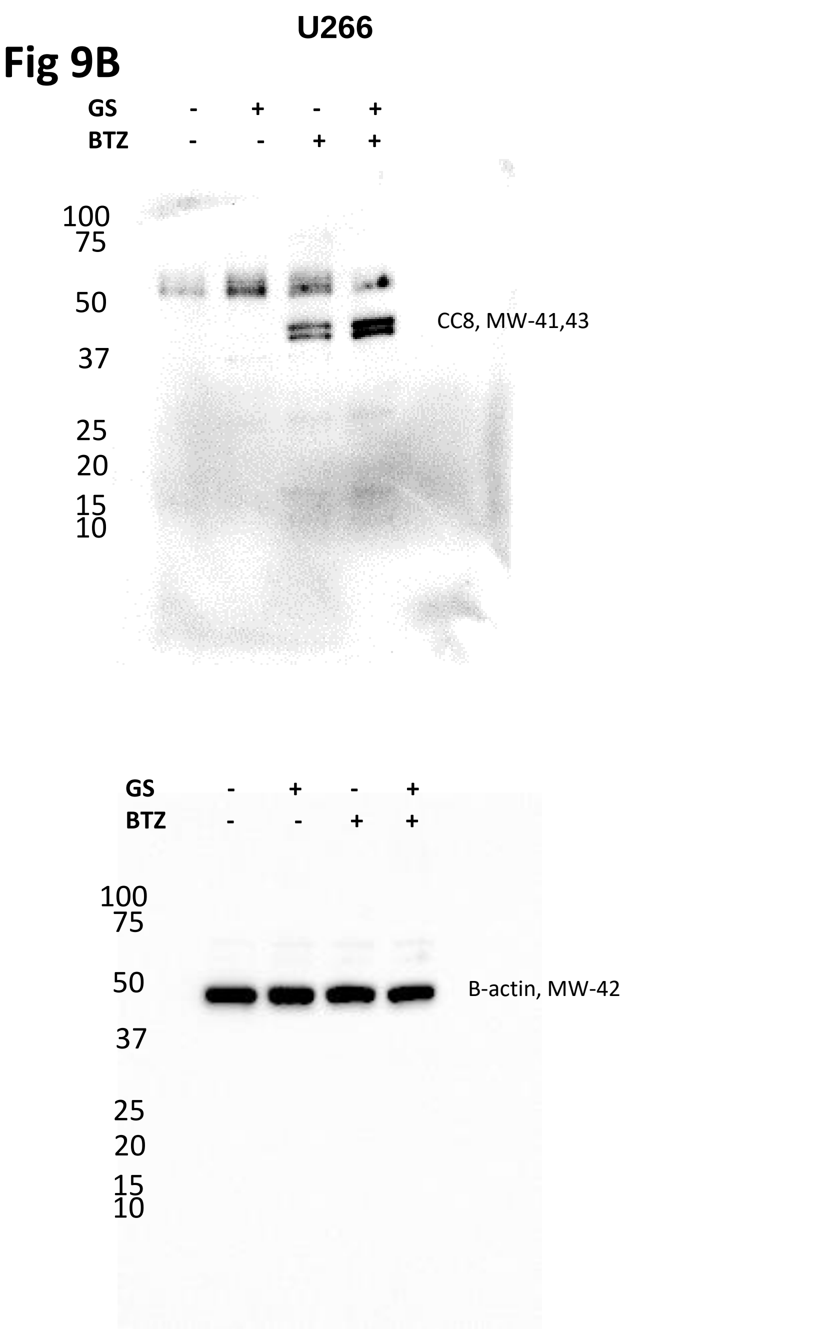

U266
Fig 9B
GS - + - +
BTZ - - + +
100
75
50
37
25
20
15
10
CC8, MW-41,43
GS - + - +
BTZ - - + +
100
75
50
37
25
20
15
10
B-actin, MW-42

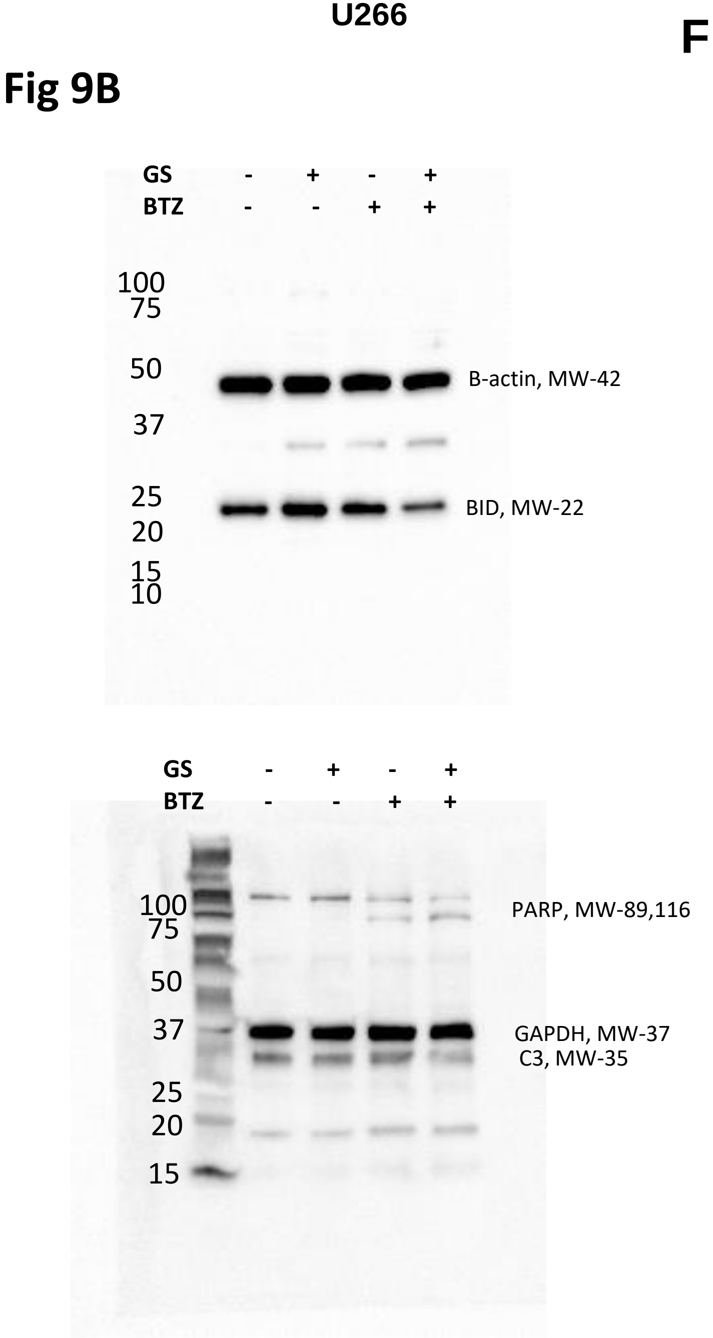

U266
Fig 9B
GS - + - +
BTZ - - + +
100
75
50
37
25
20
15
10
B-actin, MW-42
BID, MW-22
GS - + - +
BTZ - - + +
100
75
50
37
25
20
15
PARP, MW-89,116
GAPDH, MW-37
C3, MW-35

Figure 9

U266

Fig 9B

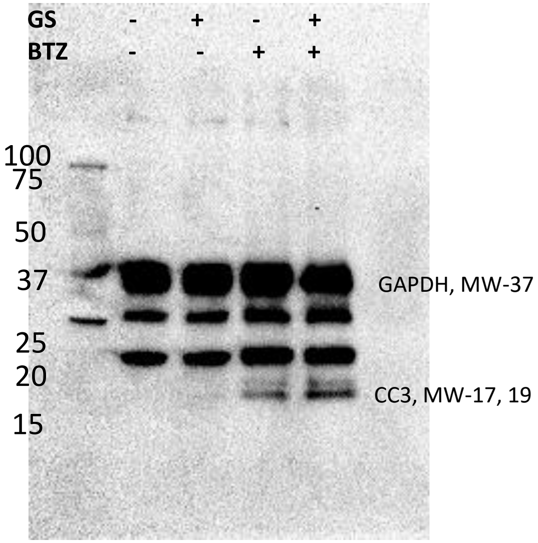

U266

Fig 9B

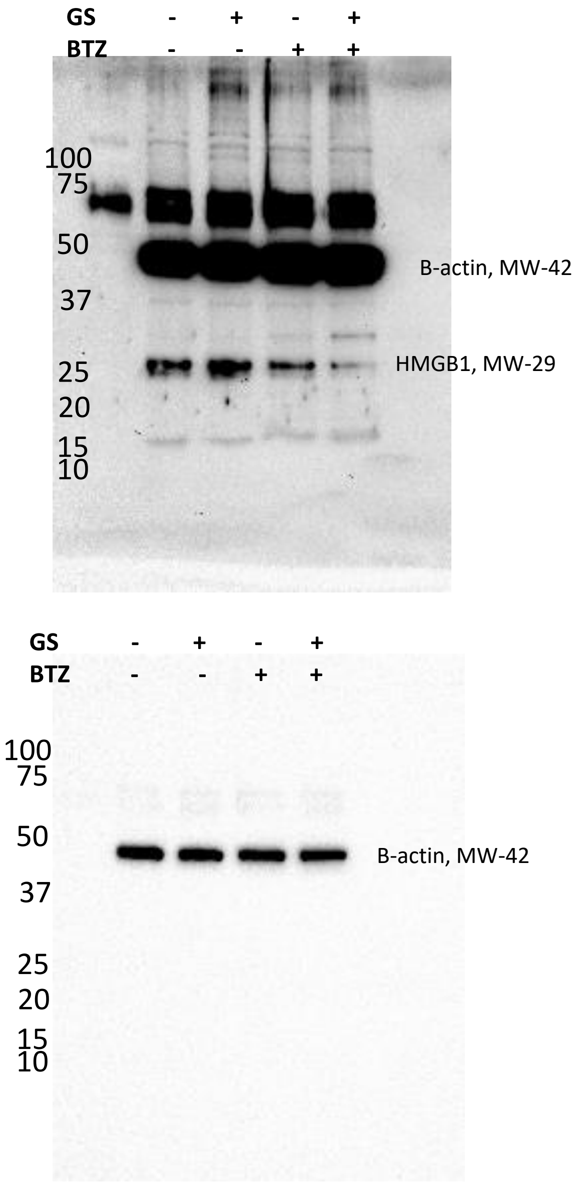

Supplementary Figure 1B

U266

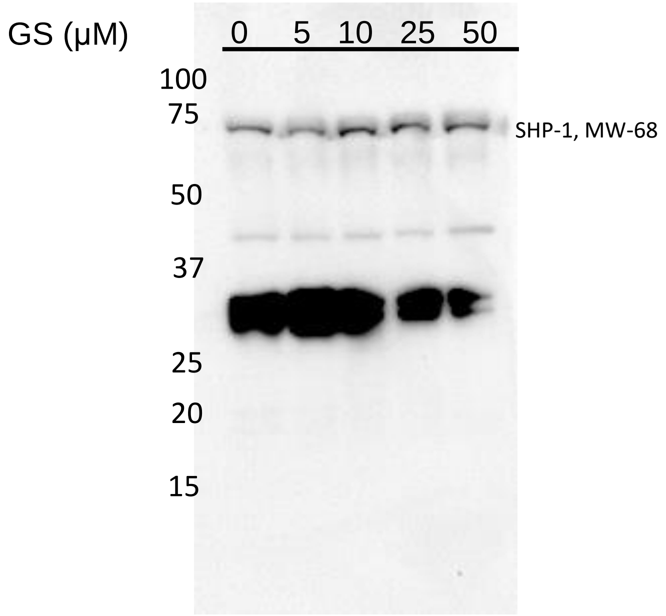

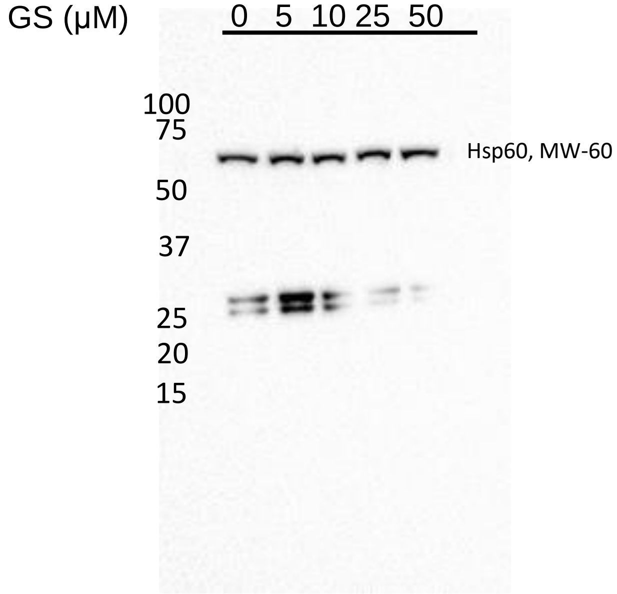

Supplement: Supplementary file 1 [file cancers-14-05621-s001.zip › file S1.pdf]
