# Supplementary material for: Guggulsterone Induces Apoptosis in Multiple Myeloma Cells by Targeting High Mobility Group Box 1 via Janus Activated Kinase/Signal Transducer and Activator of Transcription Pathway
Source: Cancers (Basel). 2022 Nov 16;14(22):5621. doi: 10.3390/cancers14225621 (PMC9688888; doi:10.3390/cancers14225621)
Supplement: Supplementary file 1 [file cancers-14-05621-s001.zip › Supplementary Figure S1.pptx]

## Slide 1
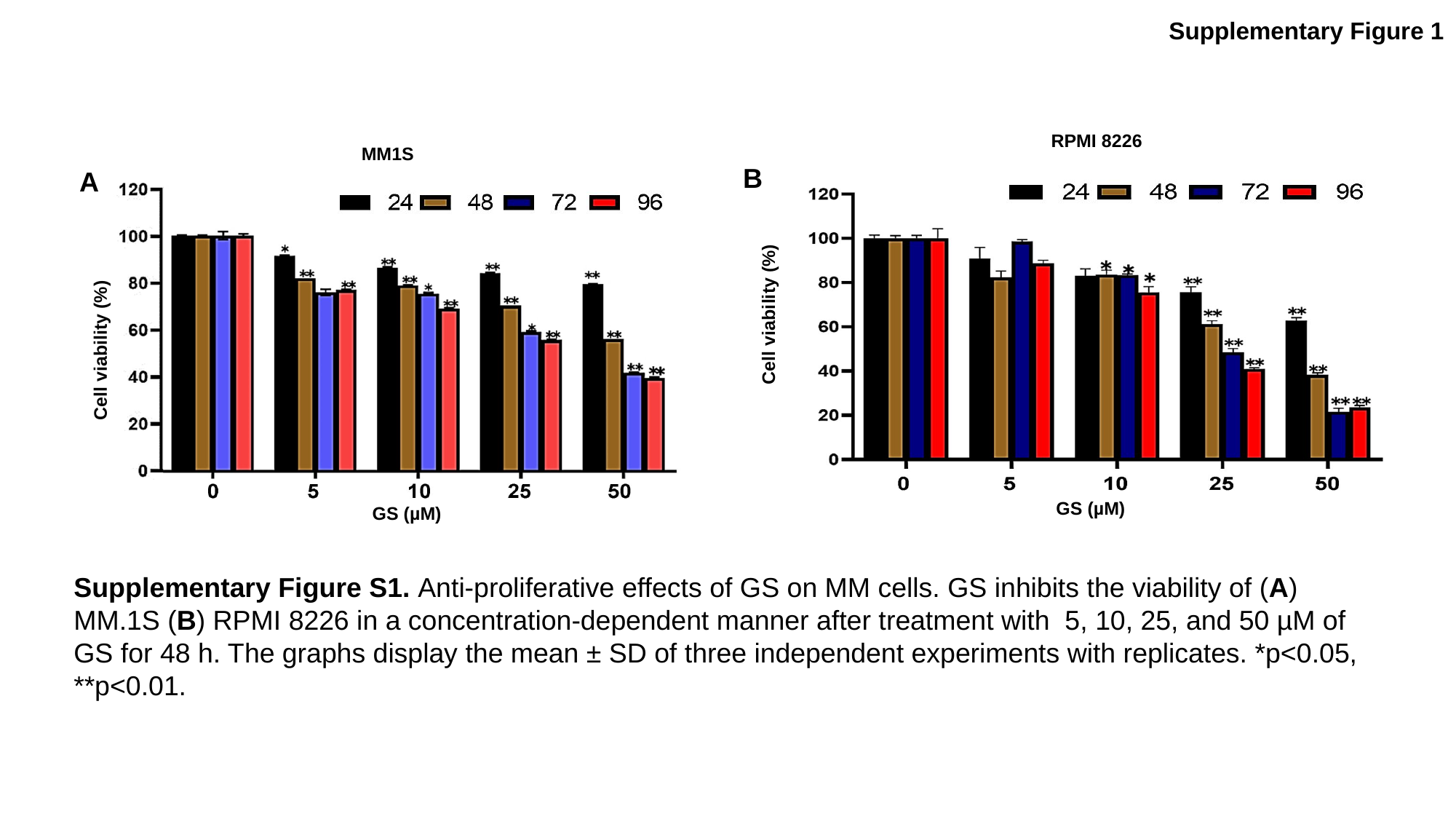

Supplementary Figure 1
RPMI 8226
B
Cell viability (%)
GS (µM)
MM1S
A
Cell viability (%)
GS (µM)
Supplementary Figure S1. Anti-proliferative effects of GS on MM cells. GS inhibits the viability of (A) MM.1S (B) RPMI 8226 in a concentration-dependent manner after treatment with 5, 10, 25, and 50 µM of GS for 48 h. The graphs display the mean ± SD of three independent experiments with replicates. *p<0.05, **p<0.01.
