# Supplementary material for: Guggulsterone Induces Apoptosis in Multiple Myeloma Cells by Targeting High Mobility Group Box 1 via Janus Activated Kinase/Signal Transducer and Activator of Transcription Pathway
Source: Cancers (Basel). 2022 Nov 16;14(22):5621. doi: 10.3390/cancers14225621 (PMC9688888; doi:10.3390/cancers14225621)
Supplement: Supplementary file 1 [file cancers-14-05621-s001.zip › Supplementary Figure S2.pptx]

## Slide 1
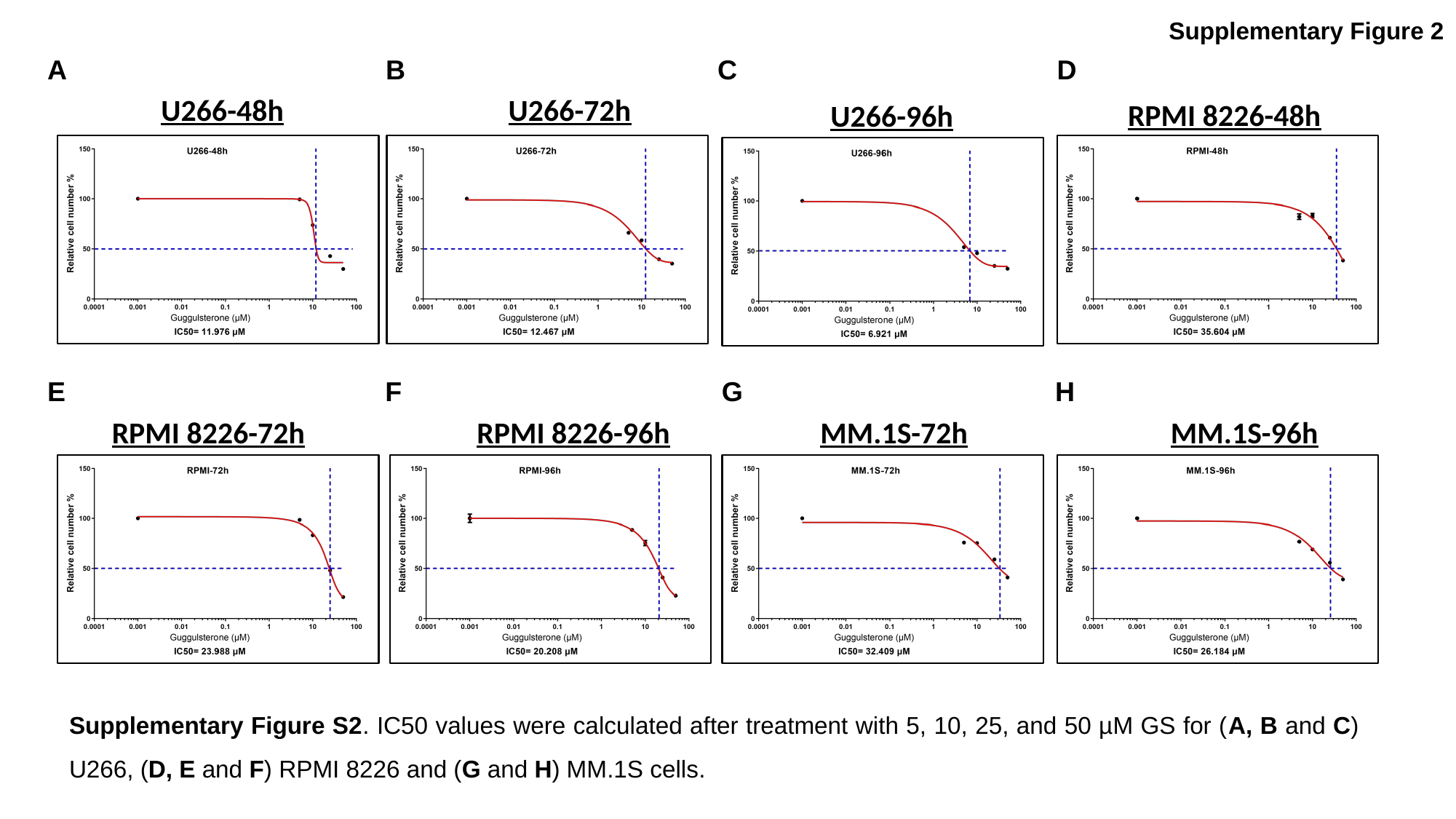

Supplementary Figure 2
A B C D
U266-48h
U266-72h
RPMI 8226-48h
U266-96h
E F G H
RPMI 8226-72h
RPMI 8226-96h
MM.1S-72h
MM.1S-96h
Supplementary Figure S2. IC50 values were calculated after treatment with 5, 10, 25, and 50 µM GS for (A, B and C) U266, (D, E and F) RPMI 8226 and (G and H) MM.1S cells.
