# Supplementary material for: Guggulsterone Induces Apoptosis in Multiple Myeloma Cells by Targeting High Mobility Group Box 1 via Janus Activated Kinase/Signal Transducer and Activator of Transcription Pathway
Source: Cancers (Basel). 2022 Nov 16;14(22):5621. doi: 10.3390/cancers14225621 (PMC9688888; doi:10.3390/cancers14225621)
Supplement: Supplementary file 1 [file cancers-14-05621-s001.zip › Supplementary Figure S3.pptx]

## Slide 1
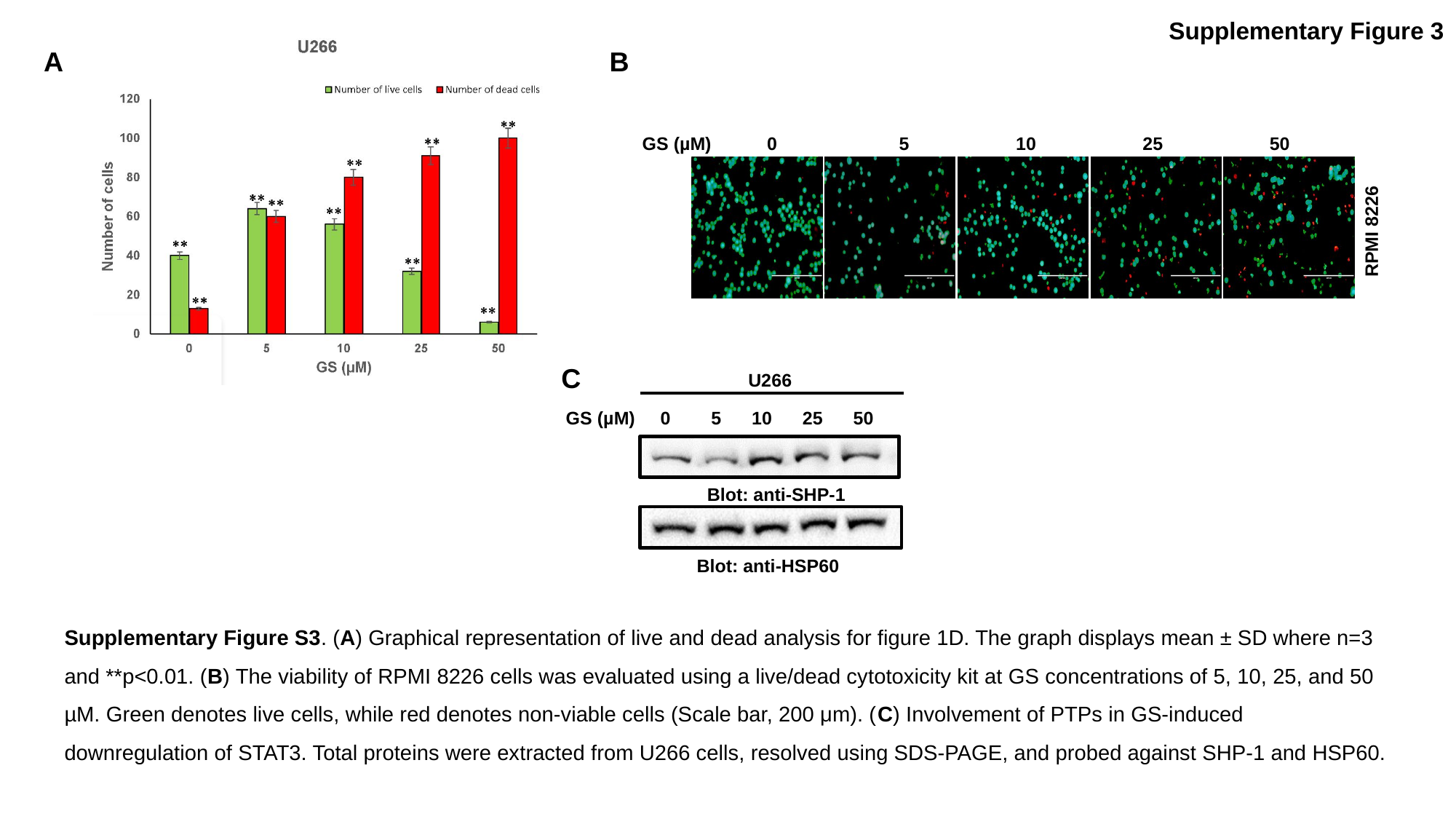

Supplementary Figure 3
A
B
GS (µM) 0 5 10 25 50
RPMI 8226
C
U266
GS (µM) 0 5 10 25 50
Blot: anti-SHP-1
Blot: anti-HSP60
Supplementary Figure S3. (A) Graphical representation of live and dead analysis for figure 1D. The graph displays mean ± SD where n=3 and **p<0.01. (B) The viability of RPMI 8226 cells was evaluated using a live/dead cytotoxicity kit at GS concentrations of 5, 10, 25, and 50 µM. Green denotes live cells, while red denotes non-viable cells (Scale bar, 200 μm). (C) Involvement of PTPs in GS-induced downregulation of STAT3. Total proteins were extracted from U266 cells, resolved using SDS-PAGE, and probed against SHP-1 and HSP60.
