# Supplementary material for: Guggulsterone Induces Apoptosis in Multiple Myeloma Cells by Targeting High Mobility Group Box 1 via Janus Activated Kinase/Signal Transducer and Activator of Transcription Pathway
Source: Cancers (Basel). 2022 Nov 16;14(22):5621. doi: 10.3390/cancers14225621 (PMC9688888; doi:10.3390/cancers14225621)
Supplement: Supplementary file 1 [file cancers-14-05621-s001.zip › Supplementary Figure S4.pptx]

## Slide 1
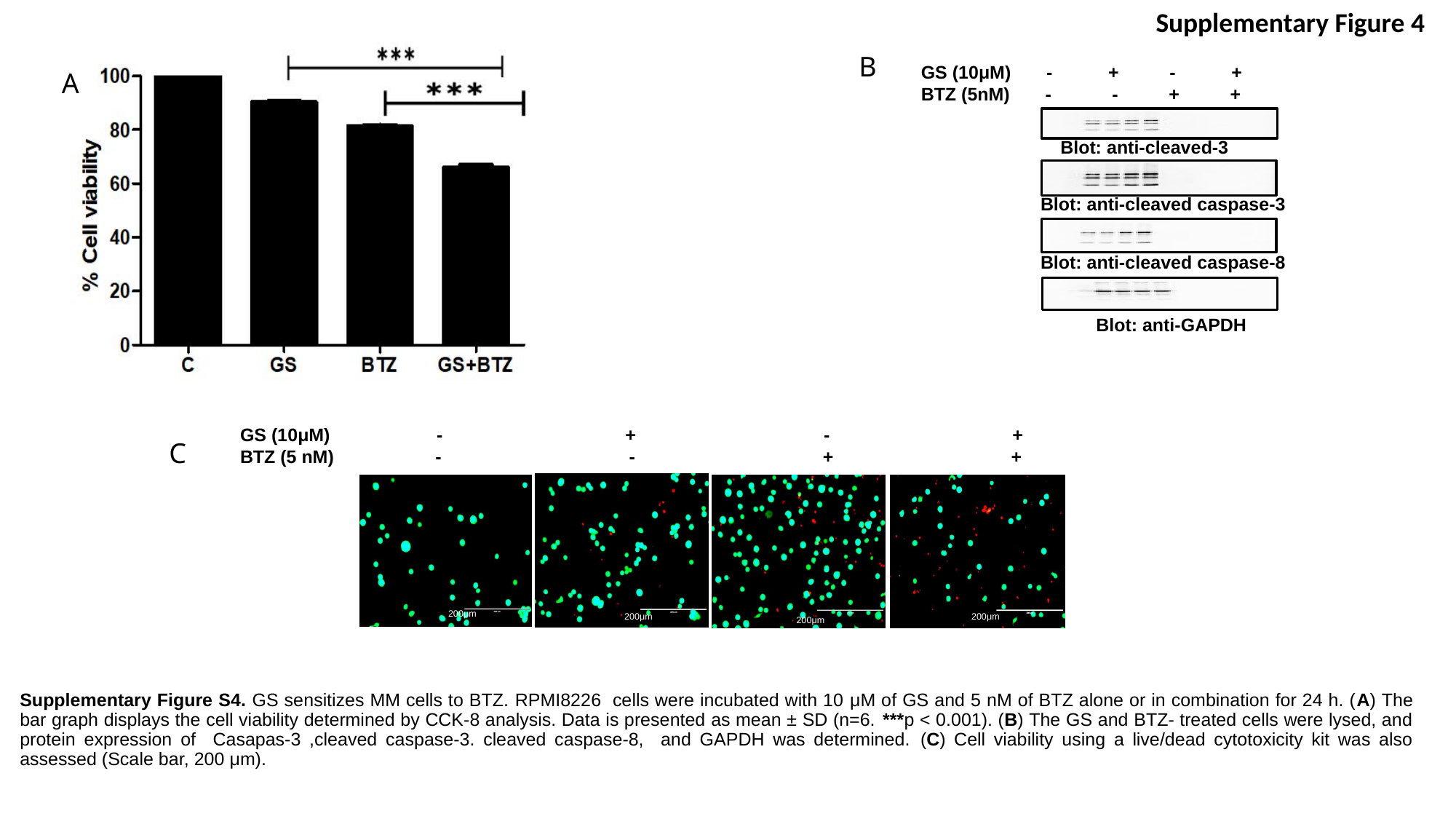

Supplementary Figure 4
B
GS (10μM) - + - +
BTZ (5nM) - - + +
Blot: anti-cleaved-3
Blot: anti-cleaved caspase-3
Blot: anti-cleaved caspase-8
 Blot: anti-GAPDH
A
GS (10μM) - + - +
BTZ (5 nM) - - + +
C
200μm
200μm
200μm
200μm
Supplementary Figure S4. GS sensitizes MM cells to BTZ. RPMI8226 cells were incubated with 10 μM of GS and 5 nM of BTZ alone or in combination for 24 h. (A) The bar graph displays the cell viability determined by CCK-8 analysis. Data is presented as mean ± SD (n=6. ***p < 0.001). (B) The GS and BTZ- treated cells were lysed, and protein expression of Casapas-3 ,cleaved caspase-3. cleaved caspase-8, and GAPDH was determined. (C) Cell viability using a live/dead cytotoxicity kit was also assessed (Scale bar, 200 μm).
